# Ca^2+^-dependent H_2_O_2_ response in roots and leaves of barley - a transcriptomic investigation

**DOI:** 10.1186/s12870-025-06248-9

**Published:** 2025-02-20

**Authors:** Sabarna Bhattacharyya, Carissa Bleker, Bastian Meier, Maya Giridhar, Elena Ulland Rodriguez, Adrian Maximilian Braun, Edgar Peiter, Ute C. Vothknecht, Fatima Chigri

**Affiliations:** 1 Institute for Cellular and Molecular Botany (IZMB), University of Bonn, Kirschallee 1, D-53115 Bonn, Germany; 2 Department of Biotechnology and Systems Biology, National Institute of Biology (NIB), Večna pot 111, Ljubljana, SI-1000 Slovenia; 3 Institute of Agricultural and Nutritional Sciences, Faculty of Natural Sciences III, Martin Luther University Halle-Wittenberg, Betty-Heimann-Str. 3, D-06120 Halle (Saale), Germany; 4 Leibniz Institute for Food Systems Biology, Technical University of Munich, Lise-Meitner- Strasse 34, D-85354 Freising, Germany

**Keywords:** ROS, Stress, RNA-Seq, Ca^2+^ signaling, Crosstalk, *Hordeum vulgare*

## Abstract

**Background:**

Ca^2+^ and H_2_O_2_ are second messengers that regulate a wide range of cellular events in response to different environmental and developmental cues. In plants, stress-induced H_2_O_2_ has been shown to initiate characteristic Ca^2+^ signatures; however, a clear picture of the molecular connection between H_2_O_2_-induced Ca^2+^ signals and H_2_O_2_-induced cellular responses is missing, particularly in cereal crops such as barley. Here, we employed RNA-seq analyses to identify transcriptome changes in roots and leaves of barley after H_2_O_2_ treatment under conditions that inhibited the formation of cytosolic Ca^2+^ transients. To that end, plasma membrane Ca^2+^ channels were blocked by LaCl_3_ application prior to stimulation of barley tissues with H_2_O_2_.

**Results:**

We examined the expression patterns of 4246 genes that had previously been shown to be differentially expressed upon H_2_O_2_ application. Here, we further compared their expression between H_2_O_2_ and LaCl_3_ + H_2_O_2_ treatment. Genes showing expression patterns different to the previous study were considered to be Ca^2+^-dependent H_2_O_2_-responsive genes. These genes, numbering 331 in leaves and 1320 in roots, could be classified in five and four clusters, respectively. Expression patterns of several genes from each cluster were confirmed by RT-qPCR. We furthermore performed a network analysis to identify potential regulatory paths from known Ca^2+^-related genes to the newly identified Ca^2+^-dependent H_2_O_2_ responsive genes, using the recently described Stress Knowledge Map. This analysis indicated several transcription factors as key points of the responses mediated by the cross-talk between H_2_O_2_ and Ca^2+^.

**Conclusion:**

Our study indicates that about 70% of the H_2_O_2_-responsive genes in barley roots require a transient increase in cytosolic Ca^2+^ concentrations for alteration in their transcript abundance, whereas in leaves, the Ca^2+^ dependency was much lower at about 33%. Targeted gene analysis and pathway modeling identified not only known components of the Ca^2+^ signaling cascade in plants but also genes that are not yet connected to stimuli-associated signaling. Potential key transcription factors identified in this study can be further analyzed in barley and other crops to ultimately disentangle the underlying mechanisms of H_2_O_2_-associated signal transduction mechanisms. This could aid breeding for improved stress resistance to optimize performance and productivity under increasing climate challenges.

**Supplementary Information:**

The online version contains supplementary material available at 10.1186/s12870-025-06248-9.

## Introduction

To withstand short-term detrimental conditions, plants have evolved complex and efficient molecular machineries to monitor and respond to environmental cues. An early plant response to many forms of stress involves reactive oxygen species (ROS) as a purposefully generated signal to modulate crucial aspects of plant growth, development, and stress adaptation [1]. ROS also constitute inevitable by-products of aerobic metabolism that under normal physiological conditions are mainly produced at a low level; however, disruption of metabolic pathways during stress often results in a dramatic increase in their rate of production [2, 3]. Hydrogen peroxide (H_2_O_2_), a very stable ROS, is generated within different cellular compartments such as chloroplasts, mitochondria, and peroxisomes, as well as extra-cellularly in the apoplast [4]. H_2_O_2_ is generated either passively by metabolic pathways such as photosynthesis, photorespiration and respiration, or produced actively by oxidases like the respiratory burst oxidase homologs (RBOHs) [3]. Also, H_2_O_2_ can be transported between different cellular compartments, cells or even tissues for the purpose of removal or accumulation, and is now considered as an important player in long-distance-signaling [5, 6].

At low levels, H_2_O_2_ can be beneficial for the plant and act as a signal transduction molecule to achieve stress tolerance; however, it can cause cellular damage and programmed cell death at higher concentrations [7]. Hence, a strict balance between production and scavenging of H_2_O_2_ is essential to prevent its accumulation to toxic levels and to ensure its function as a signaling molecule. Plants have thus evolved a complex array of enzymatic and non-enzymatic detoxification systems to adjust the H_2_O_2_ homeostasis in all subcellular compartments [8, 9]. As signaling molecule, H_2_O_2_ is involved in the regulation of various developmental and physiological processes such as root system development [10, 11], flowering [12], seed germination [13], senescence [14] and stomatal aperture [15]. Additionally, studies have uncovered key roles for H_2_O_2_ as a second messenger in the signaling pathways associated with environmental stress responses in *Arabidopsis thaliana* and crop species such as drought [16, 17], salinity [18], heat [19, 20], UV radiation [21], ozone [22], chilling [23], heavy metal [24], and pathogens [25, 26]. Various stimuli can induce increases of H_2_O_2_ levels, known as the “oxidative burst”, which is subsequently sensed and transmitted to activate downstream processes including transcriptional reprograming to elicit appropriate adaptive stress responses [27]. Moreover, H_2_O_2_ can activate other signaling cascades involving secondary messengers such as nitric oxide, phytohormones, and Ca^2+^.

Ca^2+^ also plays a pivotal role in the regulation of various developmental processes and response to environmental stresses. Changes in cytosolic free Ca^2+^ concentrations ([Ca^2+^]_cyt_) are one of the earliest cellular responses observed in plants to almost every biotic and abiotic stress that has been investigated, including salt [28, 29], cold [30, 31], drought [32–34], heat [35, 36], heavy metals [37], and pathogens [38, 39]. The transient changes in [Ca^2+^]_cyt_ are sensed and decoded by a toolkit of Ca^2+^ sensor proteins like calmodulins (CaMs), calmodulin-like proteins (CMLs), calcineurin B-like proteins (CBLs), and CBL-interacting protein kinases (CIPKs) as well as Ca^2+^-dependent protein kinases (CPKs/CDPKs) [40]. Like H_2_O_2_, Ca^2+^ signaling affects different cellular processes including regulation of gene transcription and associated downstream responses [41].

A crosstalk between Ca^2+^ and H_2_O_2_ signaling pathways has been shown in response to various abiotic and biotic stresses [42, 43]. A number of studies indicated that Ca^2+^ acts as an upstream component in H_2_O_2_ signaling by regulating H_2_O_2_ production. In plants, RBOHs possess a cytosolic N-terminal regulatory domain containing Ca^2+^-binding EF-hand motifs and Ca^2+^-dependent phosphorylation sites as targets for CPKs that are necessary for RBOH activation [44–46]. By contrast, there is also evidence that H_2_O_2_ acts as an upstream signal by inducing [Ca^2+^]_cyt_ transients involved in plant responses such as stomatal closure, programmed cell death, and other stress adaptation [47–49]. H_2_O_2_-induced Ca^2+^ release is likely due to the direct regulation of Ca^2+^-permeable channels. Annexins, cyclic nucleotide gated channels (CNGCs), and mechanosensitive ion channels (MSLs) have been proposed to function as H_2_O_2_-activated Ca^2+^ channels that mediate cellular Ca^2+^ influxes [50, 51]. In a recent study a H_2_O_2_-sensor in plants, H_2_O_2_-INDUCED CA^2+^ INCREASES 1 (HPCA1) was identified that mediates H_2_O_2_-induced activation of Ca^2+^ channels in guard cells leading to elevation in [Ca^2+^]_cyt_ and in turn initiation of stomatal closure [52]. Intriguingly, it has been shown that HPCA1 is required for systemic ROS- and Ca^2+^-mediated cell-to-cell signaling and that this includes the Ca^2+^ permeable channel MSL3 as well as the Ca^2+^ sensor CBL4 and its interacting protein kinase CIPK26 [51]. However, despite the large volume of reports and studies, it remains unclear how H_2_O_2_ and Ca^2+^ signals regulate each other, what determines the directionality of the crosstalk, and what connects both signaling pathways to achieve their synergistic response.

We thus intended to identify the contribution of cytosolic Ca^2+^ signals to H_2_O_2_-induced transcriptomic changes in leaves and roots of barley. Barley is an important global feed and food source and has been widely studied as a model for monocot crops due to its diploid nature and ease of cross-breeding [53, 54]. The effect of H_2_O_2_ on the transcriptome was recently elucidated in barley leaves and roots [55], revealing common as well as tissue-specific changes in transcript abundance of over 4000 genes including various transcription factors (TFs), genes associated with hormone pathways, and other vital functions such as photosynthesis, cell wall biogenesis, and H_2_O_2_ detoxification. It has also been shown that barley, as other plants, reacts to H_2_O_2_ application with a transient elevation in [Ca^2+^]_cyt_ [56]. For the comparative approach carried out in the current study, Ca^2+^ transients were pharmacologically inhibited by the well-known plasma membrane Ca^2+^ channel blocker LaCl_3_. RNA-seq analyses revealed that 1652 of the previously identified H_2_O_2_ responsive genes were fully or partially dependent on Ca^2+^ signals for their regulation since their differential expression was altered when the Ca^2+^ signal was inhibited by LaCl_3_. Subsequent network analyses provided testable hypotheses on the molecular mechanisms of the crosstalk between oxidative stress and Ca^2+^ signaling. Ultimately, understanding the underlying molecular processes of this crosstalk might increase our ability to improve stress resistance in barley and other crops to optimize performance and productivity under increasing climate challenges.

## Materials and methods

### Plant material, growth conditions, and stress treatment

Barley plants *(Hordeum vulgare* cultivar Golden Promise) were grown for five days in pots filled with water-soaked vermiculite in a climate-controlled growth chamber under long-day conditions with 16 h light at 20 °C and a light intensity of 120 µmol photons m^− 2^ s^− 1^ (Philips TLD 18 W of alternating 830/840 light color temperature) and 8 h darkness at 18 °C. For stress treatments, five-day-old barley seedlings were removed from the pots and incubated in ddH_2_O with or without 10 mM LaCl_3_ for one hour, briefly rinsed and then treated with ddH_2_O with or without 10 mM H_2_O_2_ for three hours. Seedlings were thoroughly rinsed before subsequent analyses.

### H_2_O_2_ staining and microscopic analyses

A modified protocol from [57] was used to stain H_2_O_2_ in barley leaves and roots with 2’,7’-dichlorodihydrofluorescein diacetate (H_2_-DCFDA; Thermo Fisher Scientific, USA). After stress treatment as described above, the seedlings were washed carefully and treated with 10 µM H_2_-DCFDA in 0.25% DMSO in the dark for one hour, followed by vacuum infiltration for 1 min in a desiccator. Approximately 5 mm segments of both tissues were mounted on a slide using tape. The fluorescence of 2’,7’-Dichlorfluorescein (DCF) was analyzed using a Leica SP8 Lightning confocal laser scanning microscope (Leica Microsystems, Germany) with an excitation wavelength of 488 nm and emission between 517 and 527 nm which was detected using a HyD Detector. Fluorescence signals were quantified in regions of interest (ROIs) using the integrated LASX software (Leica Microsystems, Germany).

### Ca^2+^ measurements using genetically encoded *APOAEQUORIN*

Effects of LaCl_3_ on Ca^2+^ signals were analysed as previously described [56]. Hv-AEQ_cyt_ plants expressing *APOAEQUORIN* were grown for five days on water-soaked vermiculite as described above, and 5 mm sections from the tip of leaves and primary roots were reconstituted in 2.5 µM coelenterazine (Carl Roth, Germany) in ddH_2_O in 96-well plates for 16 h in the dark. After reconstitution, the coelenterazine solution was replaced by ddH_2_O with or without 1 mM LaCl_3_, and samples were placed for one hour in light before measurements. Baseline luminescence was recorded for 90 s with an integration time of 1 s in a plate luminometer (Mithras LB940, Berthold Technologies, Germany) before injection of an equal volume of a 2-fold-concentrated solution of H_2_O_2_ (final concentration 10 mM). Changes in luminescence were recorded for another 600 s before the injection of a 2-fold-concentrated discharge solution (final concentration 1 M CaCl_2_ in 10% ethanol) and a subsequent recording of luminescence for 300 s. [Ca^2+^]_cyt_ was calculated as described in [48]. To calculate Δ[Ca^2+^]_cyt_, the mean of [Ca^2+^]_cyt_ derived from 10 s of baseline prior to treatment was subtracted from the maximum increase of [Ca^2+^]_cyt_ obtained after injection.

### RNA-sequencing and data analyses

After stress treatments as described above, plants were carefully washed with ddH_2_O several times before roots and leaves were separated and ground into a fine powder under liquid nitrogen using mortar and pestle. Total RNA was isolated from the tissues using the Quick-RNA miniprep Kit (ZymoResearch, USA) following the manufacturer’s instructions. The quality of RNA was assessed using a NABI Nanodrop UV/Vis Spectrophotometer (MicroDigital, South Korea). Integrity of the extracted RNA was confirmed by separation of the 28 S and 18 S rRNA bands on a 1% agarose gel.

RNA-seq was performed on three biological replicates for each treatment. Each replicate consisted of pooled material from three plants. 3’ mRNA sequencing including synthesis, labelling, and hybridization of cDNA was performed at the NGS core facility (Medical Faculty at the University of Bonn, Germany) using a NovaSeq6000 (Illumina, USA). cDNA library preparation was done using the QuantSeq protocol [58], where oligo dT priming was followed by complementary strand synthesis without any prior removal of ribosomal RNA. All further steps of data processing and alignment were performed as previously described [55]. Gene counts were approximated from the aligned files using the FeatureCounts function from the Rsubread package [59]. Differential expression analyses using the normalized counts were carried out using the DeSeq2 package [60], with default parameters for variance stabilizing transformations. The False Discovery Rate (FDR) cutoff for inclusion of data was set to 0.01. Principal Component Analyses (PCA) plots were generated with the gene counts for each sample using the princomp() function, in order to analyze and map the different variances obtained in this study. The volcano plots were made using ggplot2 and ggrepel packages of RStudio. A homology search against the genome of the model organism *A. thaliana* (TAIR 10) was performed using the Barley Reference Transcript (BaRTv1.0) dataset [61] available at www.ics.hutton.ac.uk with an E-value cutoff of 1e^− 30^. K-means clustering analyses [62, 63] was carried out using the base k-means function on RStudio with the help of pre-defined clusters determined with the help of the gap statistic method [64]. The clustering analyses were performed separately for leaf and root tissues. The clusters were then represented as heatmaps using the pheatmap function.

### Network analyses

Stress Knowledge Map is a plant molecular interaction resource, containing the Comprehensive Knowledge Network (CKN), a large, condition agnostic knowledge graph of molecular interactions in *A. thaliana* [65]. CKN was used to identify potential upstream regulators of the Ca^2+^-dependent H_2_O_2_ responsive genes. The network was first filtered to only reliable interactions (rank 0 - highest reliability, rank 1, and rank 2 edges), and GoMapMan (GMM) [66] annotations used to extract genes known to be involved in Ca^2+^ signaling (171 nodes annotated with GMM terms “30.3 - signaling.calcium”, “34.21 - transport.calcium”, or “34.22 - transport.cyclic nucleotide or calcium regulated channels”) or know to be involved in redox signalling (119 nodes annotated with GMM terms “21.1 - redox.thioredoxin”, “21.2 - redox.ascorbate and glutathione”, “21.4 - redox.glutaredoxins”, or “21.5 - redox.peroxiredoxin”). Shortest paths from the known Ca^2+^ involvement (“source”) set to *A. thaliana* homologs of the newly identified Ca^2+^-dependent H_2_O_2_ responsive genes (“target” set), with a maximum path length of three were extracted from CKN. To improve the biological plausibility of the extracted paths, we required that only a single transcriptional regulatory interaction was present in each path, and it directly regulates the target. The shortest paths were filtered to the closest source(s) per target, and merged. The same approach was taken to identify paths from the known redox related (source) set to the *A. thaliana* homologs of the Ca^2+^-independent H_2_O_2_ responsive genes. The analysis was performed in Python using Stress Knowlegde Map (SKM) tools [65], the networkX library [67], and graph-tools [68]. Results were visualised in Cytoscape [69] using the py4cytoscape library [68, 70]. Code for the network analyses is available on GitHub (see Availability of data and materials). The Cytoscape session file is available as an additional file (Additional File 1).

### cDNA synthesis and RT-qPCR

Synthesis of cDNA was carried out with 0.5–1 µg of total RNA using the ThermoFisher first strand cDNA synthesis kit with oligo-dT_18_ primers (Thermo Fisher Scientific, USA) following the manufacturer’s instructions. The cDNA synthesis reaction was terminated by heating at 70 °C for five minutes. 1:5 dilutions of the cDNAs were used for amplification, with 2 µl of the diluted cDNA added to a total reaction volume of 10 µl. RT-qPCR was carried out on a BioRad CFX 96 real-time PCR detection system (Biorad, USA) with a reaction mixture consisting of SYBR Green PCR Mix (Thermofisher Scientific, USA), forward and reverse primers (Table S1), ddH_2_O, and the template cDNA. Transcript levels were calculated using the 2^–∆∆Ct^ method [71] after normalization against *HvACTIN* and *HvGAPDH*. Data analyses, including preparation of bar graphs followed by ANOVA and Tukey’s Post-Hoc multi comparison tests, were performed using the tidyverse and agricolae packages, respectively, in RStudio. Linear regression analyses were also performed for the RT-qPCR. The base lm () function was used for the analyses. Correlation analysis was additionally carried out with the Karl Pearson method, using the cor.test () function.

## Results

### Analysis of the transcriptional effects of H_2_O_2_ and LaCl_3_ treatment in barley leaves and roots

**Fig. 1:**
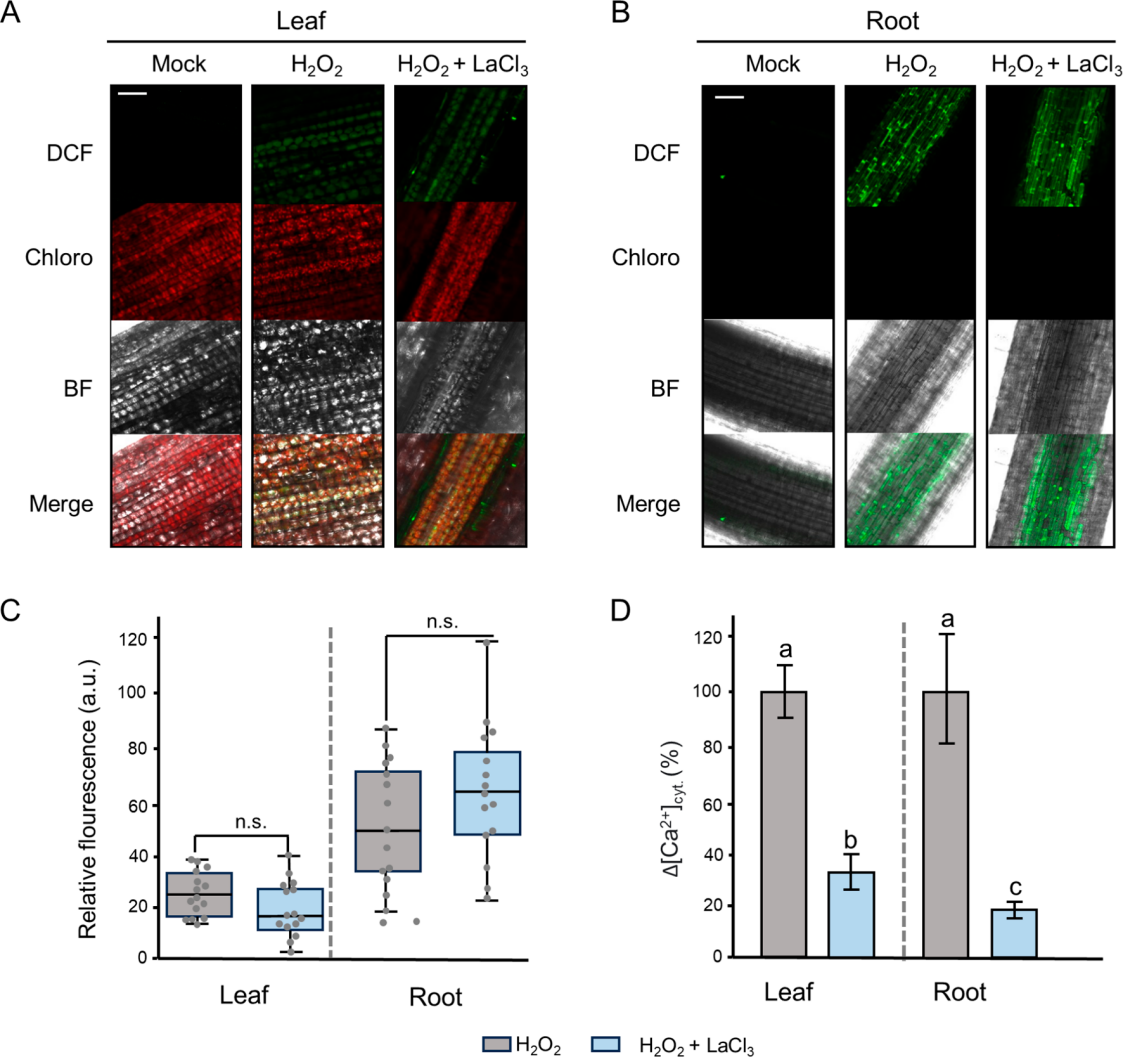
Effects of LaCl_3_ on the penetration of H_2_O_2_ and on H_2_O_2_-induced Ca^2+^ signals in barley. Plants were pre-treated either with or without 1mM LaCl_3_ before application of 10mM H_2_O_2_. For visualization of H_2_O_2_ in (**A**) leaves and (**B**) roots of barley, H_2_DCFDA staining was employed. BF: bright field, Chloro: Chlorophyll autofluorescence, DCF: Dichlorofluorescein, scale bar: 50 μm. (**C**) Quantification of relative DCF fluorescence using the LASX software. Values represent means ± SE of three independent replicates with 5 ROIs each (*n* = 15). n.s.: non-significant changes, a.u.: arbitrary units. (**D**) Inhibition of H_2_O_2_-induced Ca^2+^ signals in barley leaf and root tips under the effect of LaCl_3_. Values represent means ± SE of three biological replicates (*n* = 3). Significances were estimated with one-way ANOVA and Tukey’s Post-Hoc HSD analyses at *P* < 0.05 cutoff

In barley, it has been shown that the application of exogenous H_2_O_2_ induces increases in [Ca^2+^]_cyt_ in both leaves and roots [56]. To investigate the contribution of Ca^2+^ signaling in the H_2_O_2_-induced transcriptomic changes, we performed RNA-seq analyses under conditions that inhibited H_2_O_2_-induced Ca^2+^ transients. For that end, barley seedlings used for RNA-seq were pre-treated with the plasma membrane Ca^2+^ channel blocker LaCl_3_ before application of H_2_O_2_. Additionally, RNA-seq was also performed on plants treated solely either with LaCl_3_ or with ddH_2_O. H_2_-DCFDA staining revealed increased H_2_O_2_ levels inside both leaves and roots of barley compared to control plants and that the pre-treatment with LaCl_3_ had no effect on the H_2_O_2_ increase in both tissues (Fig. 1A-C). Furthermore, the inhibitory effect of LaCl_3_ on H_2_O_2_-induced changes in [Ca^2+^]_cyt_ was confirmed using transgenic barley reporter lines expressing the *APOAEQUORIN* reporter gene (Fig. 1D) in line with already published data [56].

**Fig. 2:**
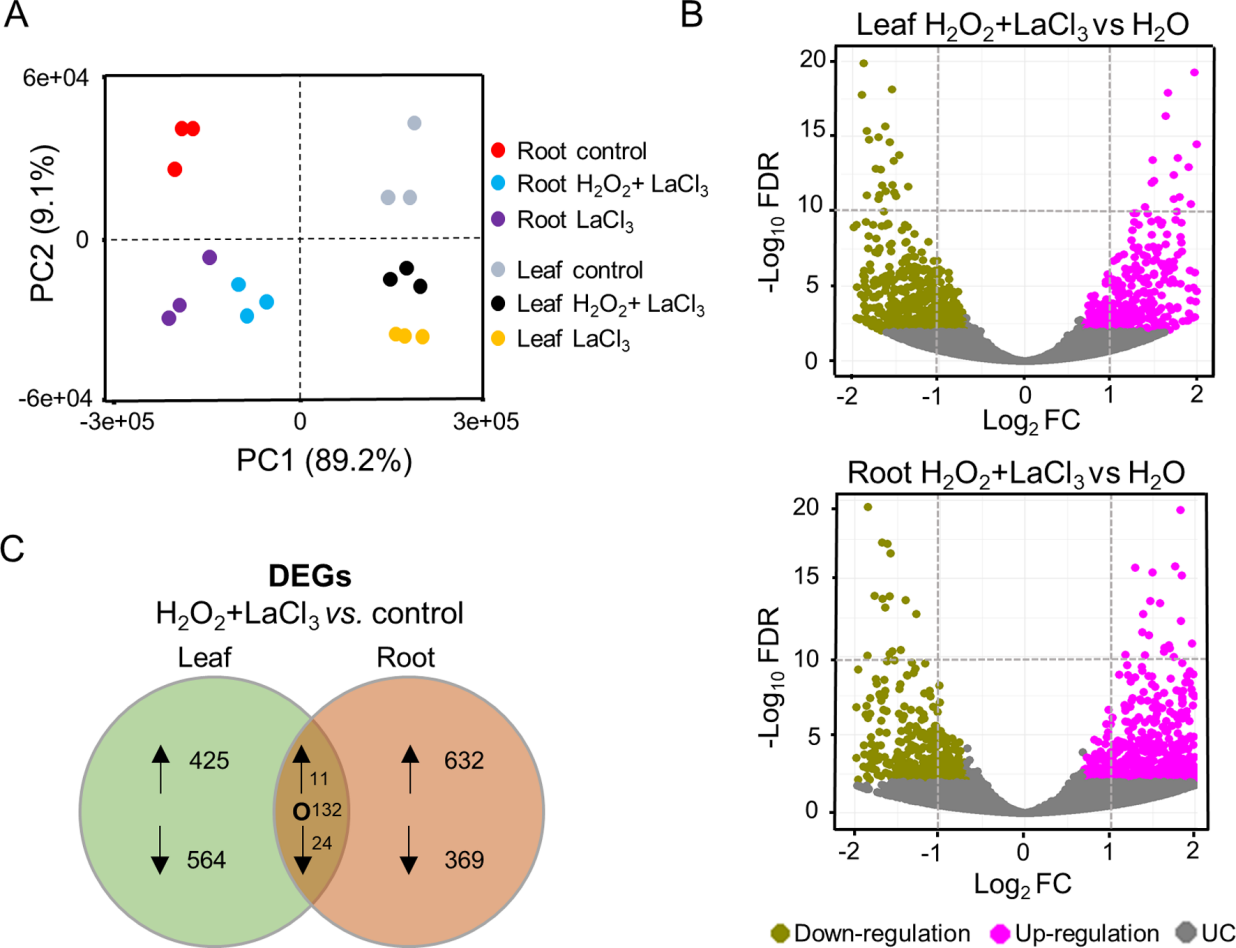
Differentially expressed genes (DEGs) in H_2_O_2_ + LaCl_3_ treated vs. control plants. (**A**) PCA plot illustrating the homogeneity of the gene counts obtained with the various treatments and tissues. PC1 (X-axis) separates the samples by tissue and PC2 (Y-axis) by treatment. (**B**) Volcano plots depicting DEGs obtained in leaves (upper panel) and roots (lower panel). The X-axis shows the fold change (log_2_FC) and the Y-axis represents the statistical significance (-log_10_FDR). DEGs (FDR < 0.01) are represented as up (magenta dots) and down (green dots) regulated, whereas genes with unchanged levels (UC) (FDR > 0.01) are indicated as grey dots. (**C**) Bubble charts representing the unique DEGs (FDR < 0.01,|log_2_FC|≥0.5) of leaves and roots, after omitting DEGs shared between the H_2_O_2_ + LaCl_3_ and the LaCl_3_ treatment. Genes found in both tissues are also indicated. Arrows indicate up (↑) and down (↓) regulation. O indicates unchanged expression

RNA-seq analysis was carried out on three biological replicates per tissue and treatment, each comprising the pooled extracted RNA from three different plants. Approximately 13–15 million raw reads were obtained and aligned against the barley reference genome (BaRTv1.0). The total alignment rate averaged from 70 to 80% across all the samples used in this study (Table 1). The aligned reads were used for differential expression analyses between the treatments and the ddH_2_O-treated control. The homogeneity of the gene counts along with their associated variance across tissues and treatments was represented as a principal component analysis (PCA) plot (Fig. 2A). The highest percentage of variance was associated with the different tissues (PC1, X-axis), with slightly lesser variance associated with the treatments (PC2, Y-axis).

**Table 1:** Summary of reads and alignment statistics. RNA-sequencing was carried out with three independent replicates. After quality control, reads were aligned against the barley reference genome (BaRTv1.0), and alignment files in bam format were then used for further processing

Sample	Replicate	Total Reads	Aligned Reads	Aligned Reads (%)
leaf LaCl_3_ + H_2_O_2_	1	13,297,596	10,033,011	75.44
	2	13,122,889	10,246,998	78.08
	3	13,201,445	10,022,100	75.91
leaf LaCl_3_	1	12,787,648	9,420,291	73.70
	2	12,541,411	9,415,802	75.10
	3	14,111,932	10,538,682	74.70
root LaCl_3_ + H_2_O_2_	1	14,455,626	10,715,747	74.12
	2	13,699,232	10,435,889	76.17
	3	13,599,945	10,166,184	74.75
root LaCl_3_	1	13,690,522	10,610,155	77.50
	2	12,208,414	9,302,812	76.20
	3	11,154,444	8,745,084	78.30

Differentially expressed genes (DEGs) between treatments and control (ddH_2_O) were defined through filtering with a cut-off of FDR < 0.01, while the other genes were considered as genes with unchanged transcript levels (UCs) (Table S2). Volcano plot analyses showed that combined H_2_O_2_ + LaCl_3_ treatment resulted in a quite similar number of up- and down-regulated genes in leaves and roots with a total number of 1006 and 1344 DEGs detected, respectively (Fig. 2B; Table S2). From these DEGs we next omitted all the genes that showed similar differential expression upon treatment with LaCl_3_ alone (Fig. S1; Table S2). Overall, this analysis identified 989 and 1001 DEGs in leaves and roots of barley, respectively, which are unique for the H_2_O_2_ + LaCl_3_ treatment (Fig. 2C, Table S2). While the overall number of DEGs was similar for both tissues, the leaves had slightly more down- and the roots considerably more up-regulated DEGs.

### Identification of Ca^2+^-dependent H_2_O_2_-responsive genes in leaves and roots of barley

**Fig. 3:**
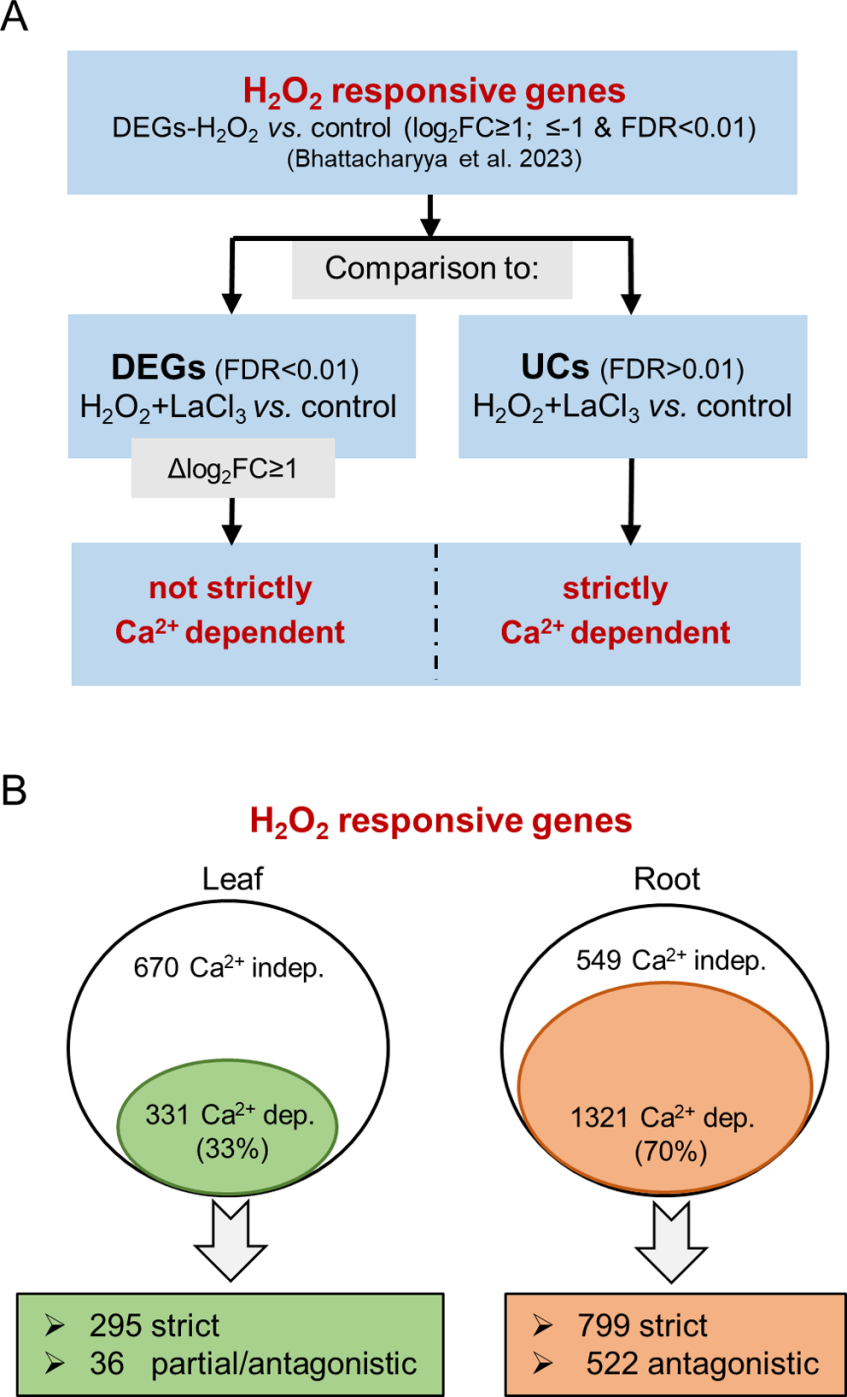
Identification of Ca^2+^-dependent H_2_O_2_-responsive genes. (**A**) Schematic representation of the bioinformatic analysis steps to identify Ca^2+^ dependent H_2_O_2_-responsive genes in leaves and roots of barley. UCs: genes with unchanged expression between H_2_O_2_ + LaCl_3_ and control. Δlog_2_FC represents the difference between log_2_FCs obtained under two conditions, i.e. H_2_O_2_ vs. control and H_2_O_2_ + LaCl_3_ vs. control. (**B**) Egg-shaped representations of the comparison between Ca^2+^-dependent and Ca^2+^-independent H_2_O_2_-responsive genes in leaves and roots of barley. The Ca^2+^-dependent genes were further divided in strict and partial/antagonistic with regards to Ca^2+^

A previous transcriptome analysis of barley had shown that 1001 and 1883 genes in leaves and roots, respectively, were differentially expressed upon H_2_O_2_ treatment [55]. These H_2_O_2_-DEGs were selected based on log_2_FC ≥ 0.5 and FDR < 0.01 and were obtained by RNA-seq of samples obtained under the same experimental conditions as in the current study. To identify those H_2_O_2_-DEGs that depend on the H_2_O_2_-induced Ca^2+^ signals for their differential regulation, a comparative analysis between the transcriptomes in response to H_2_O_2_ [previously published data, 55] and to H_2_O_2_ + LaCl_3_ was performed. More precisely, we selected those DEGs from the H_2_O_2_ treatment that either showed an unchanged expression (UCs) under H_2_O_2_ + LaCl_3_ treatment or which were DEGs under both treatments but their expression level differed significantly (Δlog_2_FC ≥ 1; corresponding to a fold change difference ≥ 2) when H_2_O_2_ treatment was compared to H_2_O_2_ + LaCl_3_ treatment (Fig. 3A). Δlog_2_FC thus represents the difference between log_2_FCs obtained under two conditions, i.e., H_2_O_2_ vs. H_2_O and H_2_O_2_ + LaCl_3_ vs. H_2_O.

All in all, about 33% and 70% of the H_2_O_2_-responsive genes in leaves and roots, respectively, were considered as Ca^2+^-dependent H_2_O_2_-responsive genes in barley (Fig. 3B). Of those, 295 genes in leaves and 799 genes in roots showed a strict dependency (DEGs-H_2_O_2_ vs. UCs-H_2_O_2_ + LaCl_3_) on Ca^2+^ signals (Fig. 3B; Table S3 and S4). 36 genes in leaves and 522 genes in roots were either partially dependent on Ca^2+^ signals (altered up- or down-regulation levels), or even displayed a counter-regulation from up to down or vice versa.

### GO analyses of Ca^2+^-dependent H_2_O_2_-responsive genes

**Fig. 4:**
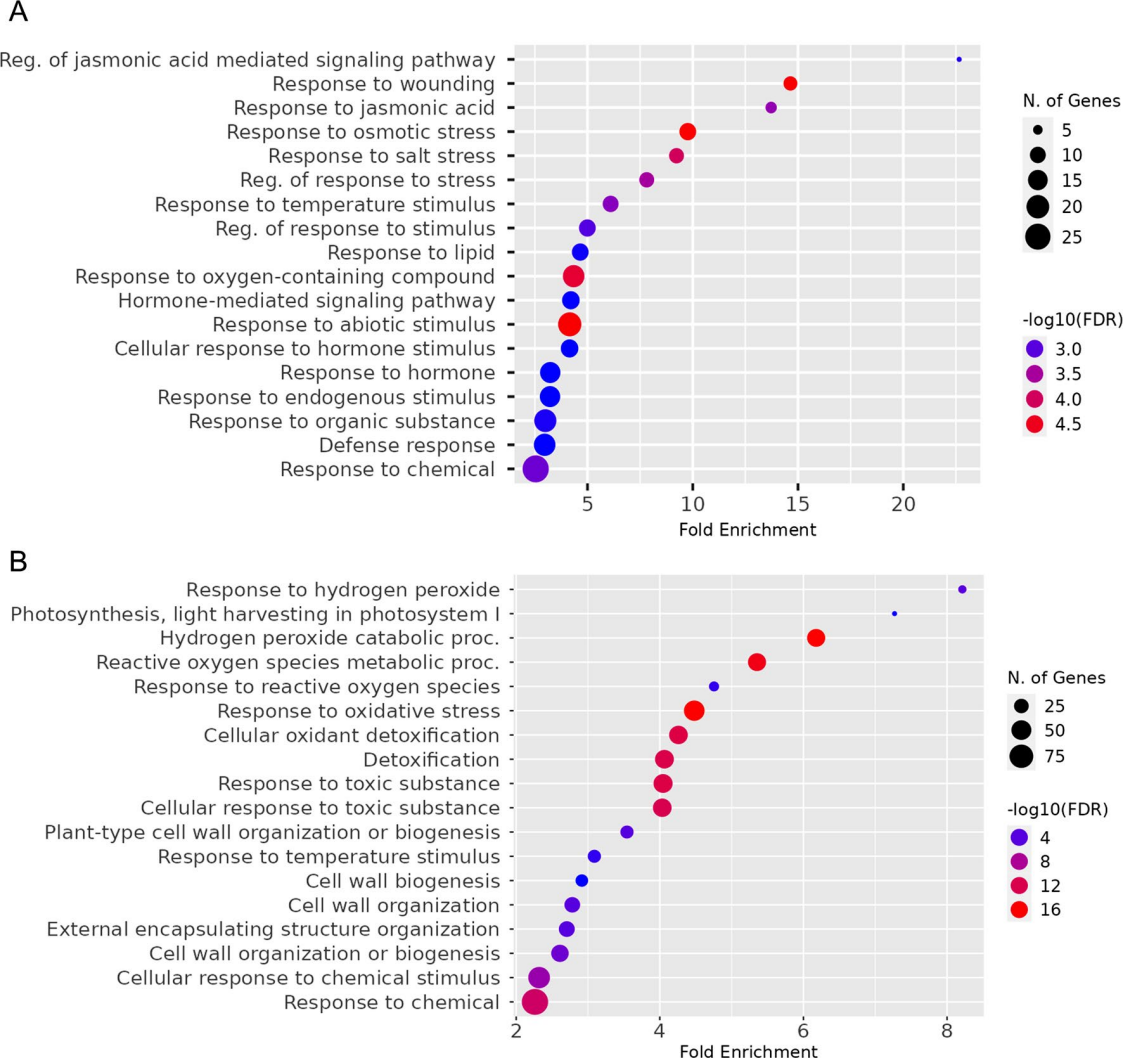
Gene ontology enrichment analysis of Ca^2+^-dependent H_2_O_2_-responsive genes. The diagrams of enriched GO terms indicate total number of genes associated with various biological processes and their fold enrichment (relative to their overall occurrence in the genome) in (**A**) leaves and (**B**) roots of barley

GO enrichment analyses were performed with the obtained Ca^2+^-dependent H_2_O_2_-responsive genes in leaves and roots of barley (Fig. 4). In leaves, the top biological terms were related to jasmonate (JA) signaling and wounding. Further enrichment was observed for terms related to abiotic stresses in general and salt, osmotic stress, and temperature in particular. Further GO terms were related to hormones and oxygen-containing compounds (Fig. 4A). By contrast, the root gene set yielded mostly GO terms associated with ROS/H_2_O_2_ response and metabolism, response to oxidative stress, and detoxification but also to cell wall biogenesis and organisation (Fig. 4B).

### Clustering analysis of Ca^2+^-dependent H_2_O_2_-responsive genes

**Fig. 5:**
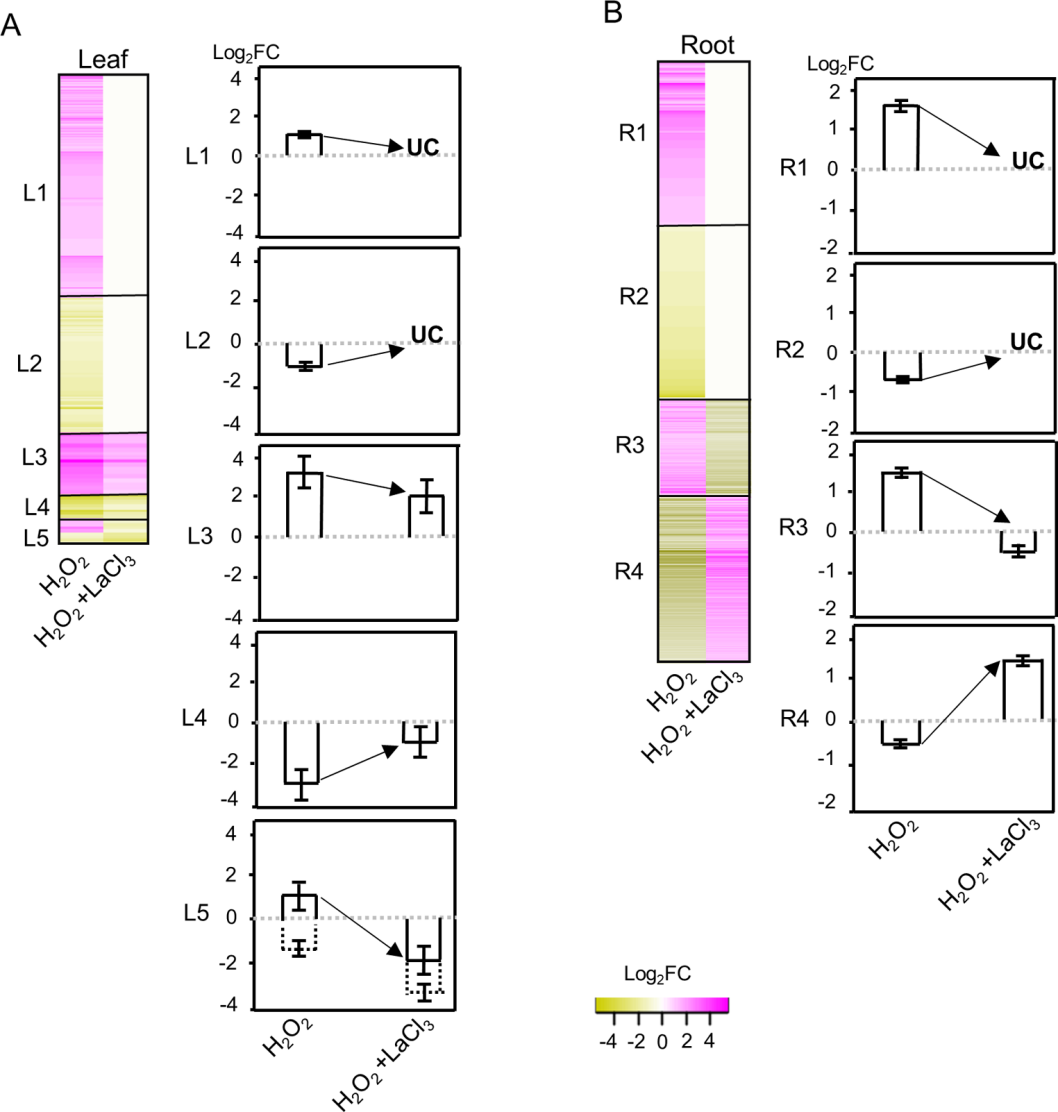
Clustering analysis of the Ca^2+^-dependent H_2_O_2_-responsive genes. Gene clustering was used to group the Ca^2+^-dependent H_2_O_2_-responsive genes with similar expression patterns. The results provided five clusters in leaves (**A**) and four clusters in roots (**B**). Left panels of each subpart represent the heatmap of the genes in the clusters, and the right panel shows a bar chart representation of the mean ± SE of the log_2_FC of the genes in each cluster. UC: genes with unchanged expression between H_2_O_2_ + LaCl_3_ and control

Clustering analysis of the Ca^2+^-dependent H_2_O_2_-responsive genes provided five clusters, L1-L5, for leaves and four clusters, R1-R4, for roots (Fig. 5, Fig. S2). In leaves, cluster L1 and L2 comprise genes which were up- and down-regulated under H_2_O_2_, respectively, however, in the presence of H_2_O_2_ + LaCl_3_ their expression level was unchanged compared to control conditions (Fig. 5A, Table S3). This indicates a strict dependence of their response to H_2_O_2_ on Ca^2+^ signals. The genes in cluster L3 and L4 showed a reduced up- and down-regulation in response to H_2_O_2_, respectively, when the Ca^2+^ transient was blocked by LaCl_3_, however, transcript levels were still significantly higher or lower compared to the control. Thus, cluster L3 and L4 represent H_2_O_2_-responsive genes with partial dependence on Ca^2+^. Cluster L5 contains H_2_O_2_-responsive genes that went from up- to down-regulation upon inhibition of the Ca^2+^ transient but also three genes for which their down-regulation was enhanced. Remarkably, in roots cluster R1 and R2 represent many genes with a strict dependence on the Ca^2+^ transient for their up- or down-regulation, respectively, however, in contrast to leaves, no partial up- and down-regulation was observed. Instead, clusters R3 and R4 comprise many H_2_O_2_-responsive genes which upon inhibition of the Ca^2+^ signal went from up- to down-regulation and vice versa (Fig. 5B, Table S4).

**Fig. 6:**
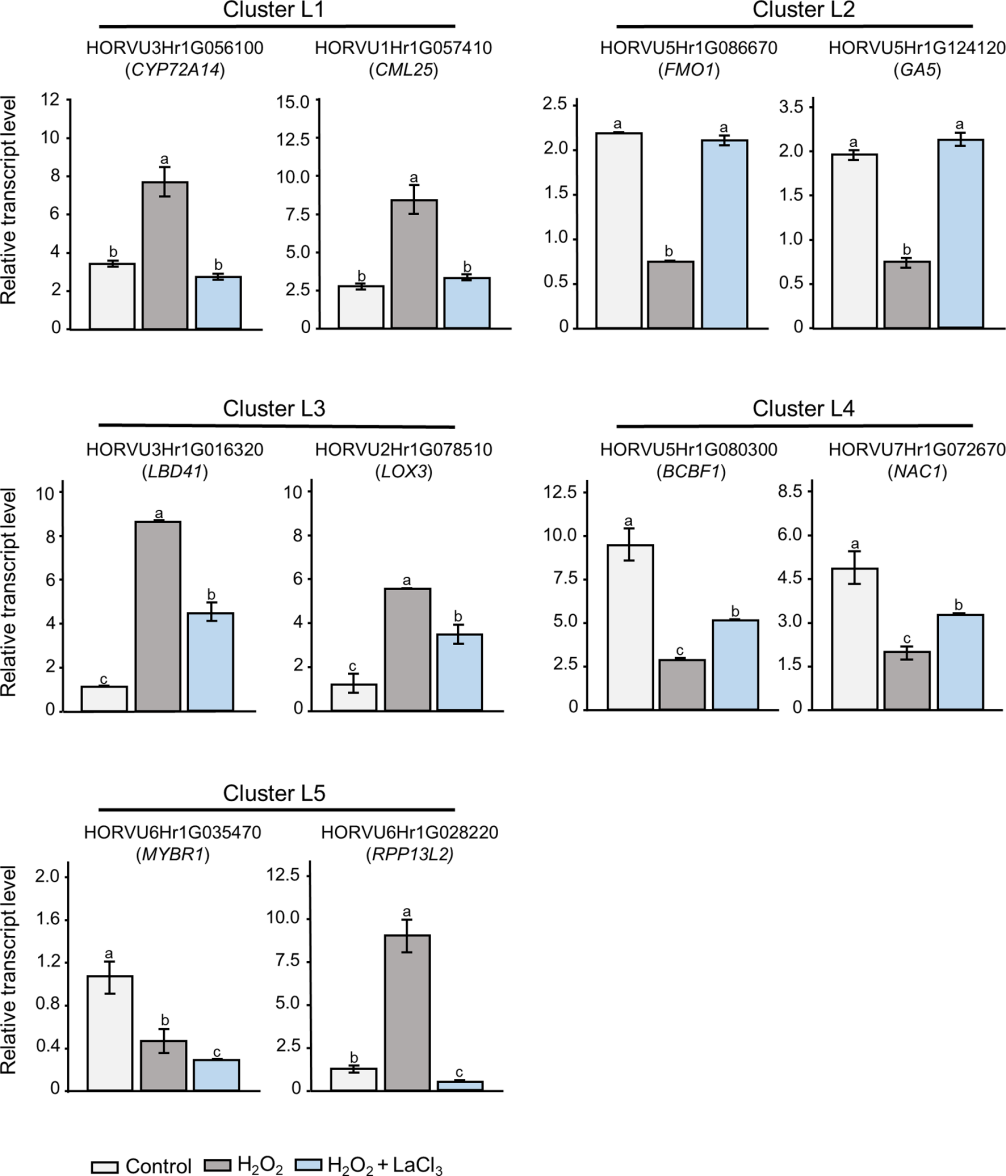
RT-qPCR analyses of transcript levels in leaves. Two Ca^2+^-dependent H_2_O_2_-responsive genes from each leaf cluster were randomly selected. Data represent mean ± SE of three independent biological replicates and two technical repeats (*n* = 3). The transcript levels were normalized to the reference genes *HvACTIN* and *HvGAPDH*. Statistical significances were obtained using one-way ANOVA and Tukey’s Post-Hoc HSD test (*P* < 0.05). The letters represent different levels of significance. Orthologous genes in Arabidopsis are indicated in brackets

**Fig. 7:**
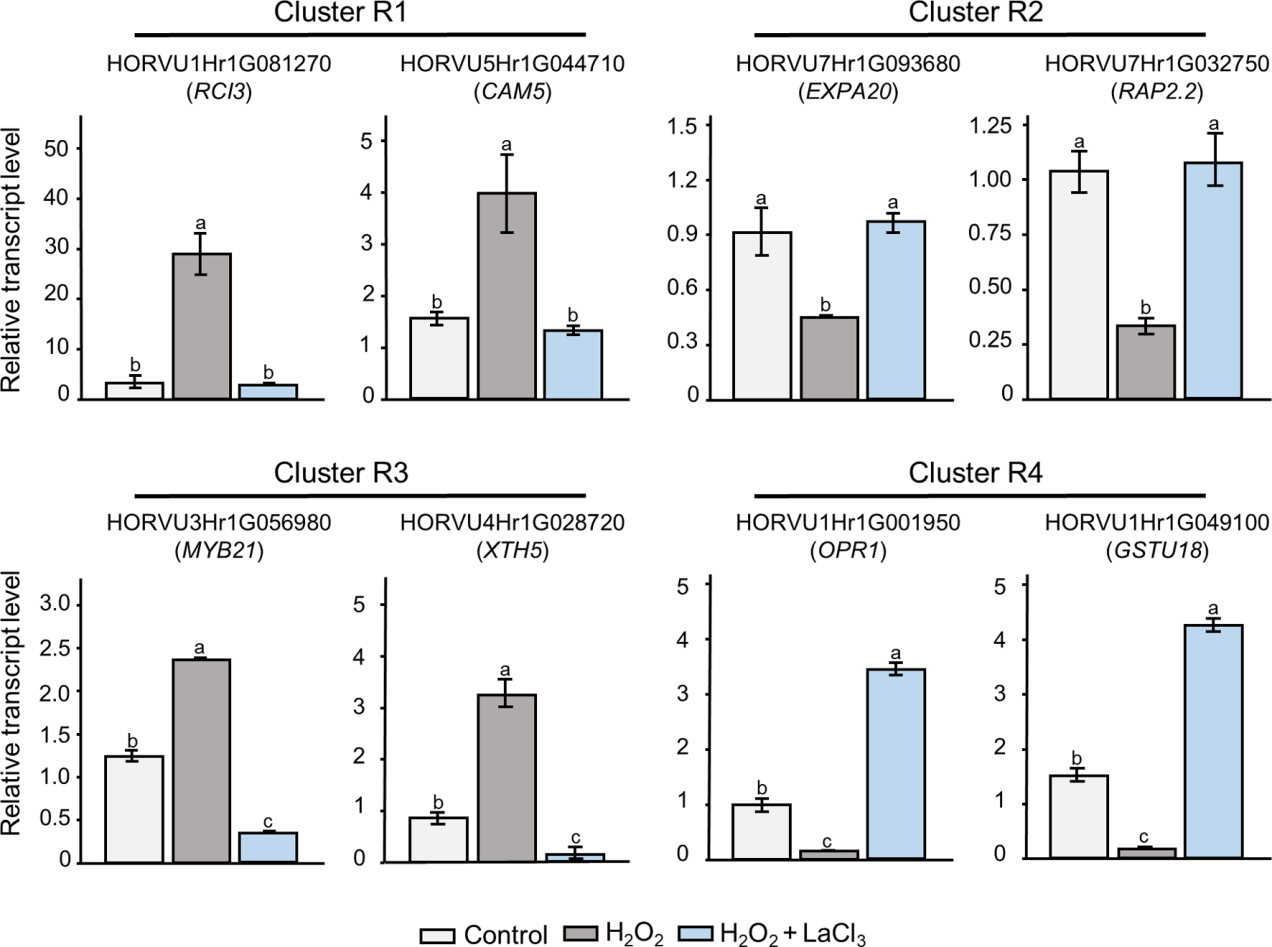
RT-qPCR analyses of transcript levels in roots. Two Ca^2+^-dependent H_2_O_2_-responsive genes from each root cluster were randomly selected. Data represent mean ± SE of three independent biological replicates and two technical repeats (*n* = 3). The transcript levels were normalized to the reference genes *HvACTIN* and *HvGAPDH*. Statistical significances were obtained using one-way ANOVA and Tukey’s Post-Hoc HSD test (*P* < 0.05). The letters represent different levels of significance. Orthologous genes in Arabidopsis are indicated in brackets

To verify the accuracy of the RNA-seq data and clustering analysis, the expression levels of two randomly selected genes from each cluster were re-evaluated by RT-qPCR (Figs. 6 and 7). For all candidate genes tested, the transcript levels determined by RT-qPCR showed similar trends as observed in the RNA-seq data. Linear regression analysis showed a correlation coefficient of > 0.7, indicating a positive correlation between RT-qPCR and RNA-seq data for all treatments and tissues (Fig. S3).

#### Cluster L1

Cluster L1 (up-regulation is strictly dependent on a Ca^2+^ signal) has a total of 196 genes, over 20 of which encode members of TF families (Table S3). Several of these TFs belong to the AP2/ERF (APETALA2/ethylene response factor) family, which has been associated with a wide variety of environmental stresses including hypoxia, cold, oxidative, and flooding stress not only in Arabidopsis but also in other plant species [72, 73]. Originally associated with ethylene signaling, AP2/ERF TFs have also been connected to other hormones like abscisic acid (ABA), gibberellic acid (GA), and cytokinin [74]. Genes associated with these hormones were also found in this cluster. Other important TFs in cluster L1 belong to the WRKY, NAC, and F-BOX domain-containing TF families. These TF families have been shown to function ubiquitously in a variety of abiotic and biotic stimuli by intercepting the ROS signaling [75–77]. Cluster L1 furthermore contains several genes related to Ca^2+^ signaling such as orthologs of genes encoding the calmodulin-like proteins AtCML11, AtCML25, or OsCML26 (LOC_Os12g01400.1), as well as AtCIPK1 (CBL-interacting protein kinase 1). It furthermore includes genes coding for members of the MAPK (mitogen activated protein kinase) and MAPKKK (MAPK kinase kinase) family. With regard to hormone signaling, genes found in cluster L1 encode negative regulators of the JA pathway including proteins involved in the degradation of the biologically active form of jasmonate, JA-Ile [78, 79]. Genes encoding proteins involved in catabolic function were also found for GA, cytokinin and ABA. We furthermore identified three auxin responsive genes, only one of which has an ortholog in Arabidopsis (*AtIAA22)*.

#### Cluster L2

Cluster L2 (down-regulation is strictly dependent on a Ca^2+^ signal) comprises a total of 99 genes. It also includes genes coding for various TFs of the AP2/ERF, WRKY, OVATE, or F-BOX families (Table S3). The AP2/ERF TFs were orthologs of AtERF1 which has been associated with both JA and ethylene signaling [80], and AtRAV2 which has been proposed to be involved in touch stimuli induced signaling [81]. Additionally, several genes encoding kinases associated with signal transduction events were identified including orthologs of the cysteine receptor kinase 28 (AtCRK28), which was associated with ROS-related stress responses [82]. Cluster L2 includes three genes encoding class III plant peroxidases, particularly orthologs of AtPRX52 and AtPRX72 [83]. Interestingly, L2 was the only cluster in leaves that includes a group of genes encoding transport proteins, such as orthologs of the ABC domain containing JA/JA-Ile transporter AtABCG16/JAT1 [84] and of AZA-RESISTANT GUANINE 2 (AtAZG2), a member of the AZG purine transporter family that has been shown to function in transportation of cytokinin [85]. Additionally, this cluster contains a number of other genes that play important roles in different stress pathways in plants such as orthologs of the *FLAVIN MONO-OXYGENASE* 1 (*AtFMO1*), which is positioned downstream of SA induced Systemic Acquired Resistance (SAR) and related signaling pathways [86] and has also been associated with AtCDPK5 a target of Ca^2+^ signals [87, 88].

#### Cluster L3

Cluster L3 (up-regulation is partially dependent on a Ca^2+^ signal) consists of 16 genes, most of which have no functional annotation and only six have a clear ortholog in Arabidopsis (Table S3). Of these genes, one encodes an ortholog of the TF AtLBD41, a class IIA LBD protein that was previously identified in relation to low-oxygen endurance or high-light-induced increase in H_2_O_2_ in Arabidopsis [89, 90] as well as flooding response in soybean [91]. Another one encodes an ortholog of the 13 S-lipoxygenase 3 (AtLOX3), an enzymes that catalyze the first step in the biosynthesis of JA [92]. LOX3 was shown to play an important role in vegetative growth restriction after wounding [93], parasitic nematode infection [94], and salt stress [95], responses all of which include H_2_O_2_ and Ca^2+^ signaling.

#### Cluster L4

Cluster L4 (down-regulation is partially dependent on Ca^2+^ signal) comprises only 10 genes, Similar to cluster L3 many have no assigned function and only three have known orthologs in Arabidopsis (Table S4). Three TFs were found including HORVU3Hr1G010190, which is a different ortholog of AtRAV2 than the one found in cluster L2. Thus, RAV2-encoding genes show both strict and partial dependence on Ca^*2+*^ in their H_2_O_2_-induced down-regulation. In this cluster we also found the gene *HORVU1Hr1G063780*, which is an ortholog of *AtGA20OX2*, which plays an important role in the rate-limiting steps of GA biosynthesis [96]. The GA20 oxidases, AtGA20OX1 and 2 are supposed to have a partially redundant function; however, we found the barley ortholog of *AtGA20OX1* within the up-regulated genes (in cluster L1).

#### Cluster L5

Cluster L5 combines genes with two different types of regulation pattern. Three of the 10 genes showed enhanced down-regulation when Ca^2+^ signals were inhibited by LaCl_3_. The other seven displayed counter-regulation going from up-regulation by H_2_O_2_ to down-regulation under combined H_2_O_2_ + LaCl_3_ treatment. For only five genes an Arabidopsis ortholog and thus a potential function was identified (Table S3) and none of the genes in cluster L5 have so far been linked to H_2_O_2_ or Ca^2+^ signaling. One gene with enhanced down-regulation encodes an ortholog of AtMYBR1, also called MYB44, a TF that has been shown to negatively regulate ABA signaling by interacting with the nuclear ABA receptor PYR1-LIKE 8 [97]. It has also been associated with other hormone responses, i.e. to JA and SA [98].

Cluster L5 combines genes with two different types of regulation pattern. Three of the 10 genes showed enhanced down-regulation when Ca^2+^ signals were inhibited by LaCl_3_. The other seven displayed counter-regulation going from up-regulation by H_2_O_2_ to down-regulation under combined H_2_O_2_ + LaCl_3_ treatment. For only five genes an Arabidopsis ortholog and thus a potential function was identified (Table S3) and none of the genes in cluster L5 have so far been linked to H_2_O_2_ or Ca^2+^ signaling. One gene with enhanced down-regulation encodes an ortholog of AtMYBR1, also called MYB44, a TF that has been shown to negatively regulate ABA signaling by interacting with the nuclear ABA receptor PYR1-LIKE 8 [97]. It has also been associated with other hormone responses, i.e. to JA and SA [98].

Cluster L5 combines genes with two different types of regulation pattern. Three of the 10 genes showed enhanced down-regulation when Ca^2+^ signals were inhibited by LaCl_3_. The other seven displayed counter-regulation going from up-regulation by H_2_O_2_ to down-regulation under combined H_2_O_2_ + LaCl_3_ treatment. For only five genes an Arabidopsis ortholog and thus a potential function was identified (Table S3) and none of the genes in cluster L5 have so far been linked to H_2_O_2_ or Ca^2+^ signaling. One gene with enhanced down-regulation encodes an ortholog of AtMYBR1, also called MYB44, a TF that has been shown to negatively regulate ABA signaling by interacting with the nuclear ABA receptor PYR1-LIKE 8 [97]. It has also been associated with other hormone responses, i.e. to JA and SA [98].

#### Cluster R1

Cluster R1 (up-regulation is strictly dependent on a Ca^2+^ signal) contains a total of 389 genes, including several TFs mostly belonging to sub-families like AP2/ERF, WRKY, MYB, OVATE, bHLH, HOMEOBOX, F-BOX, GATA, and LEA (Table S4). Cluster R1 also contains genes encoding proteins related to glutathione metabolism and other forms of detoxification. By far the largest functional group are anti-oxidant enzymes with the majority being class III plant type peroxidases. Nine of these encode different barley orthologs of AtRCI3 and seven include orhtologs to the secretory peroxidase AtPRX39 both of which has been associated with cold stress and tolerance [99, 100]. Also, genes related to Ca^2+^ signaling were identified such as orthologs of *AtCAM5* [101] and the Ca^2+^-dependent NADPH oxidase *RBOHD* [45, 102], *AtCPK5* [103], and *AtMPK9*, a MAP kinase shown to positively regulate ROS-mediated ABA signaling downstream of Ca^2+^ signals [104]. Other kinases include orthologs of the cytoplasmic histidine kinase *AtAHK5*, the mutation of which leads to reduced stomatal closure in response to H_2_O_2_ [105] The gene *HORVU5Hr1G046020* encodes an ortholog of *AtPBL8*, a member of the subfamily VII of receptor-like cytoplasmic kinases (RLCK), other members of which were found in all root clusters and in leaf clusters L1 and L2. Several RLCKs play a role in pattern-triggered immune signaling, and the higher order mutant *atpbl8/16/17* showed increased flg22-triggered H_2_O_2_ generation [106].

#### Cluster R2

Cluster R2 (down-regulation is strictly dependent on a Ca^2+^ signal) is the largest cluster with 410 genes (Table S4). Again, a number of TFs belonging to different families were found in this cluster, including an ortholog of *AtERF1*, albeit a different one to the ortholog found in cluster L2. Similar to cluster R1, this cluster also contains genes encoding proteins involved in ROS metabolism and detoxification, such as another ortholog of AtPRX52. The cluster R2 contains several genes coding for proteins with Ca^2+^-binding EF-hand domains, one of them being an ortholog of AtCML39. Interestingly in this cluster we found six genes related to photosynthesis, encoding orthologs of the Arabidopsis chlorophyll-binding proteins of the LHCA and LHCB type as well as AtPSB28 and AtPSAK. Cluster R2 also comprises orthologs of several genes involved in hormonal signaling.

#### Cluster R3

Cluster R3 (counter-regulation from up to down) contains 128 genes. As in most clusters, we found genes belonging to major TF families (Table S4). We also found two peroxidases, orthologous of Arabidopsis *AtPRX71* and *AtRCI3*, the ortholog of TPR like thioredoxin *AtTTL1*, and genes associated with various aspects of hormone signaling. Additionally, several components of Ca^2+^ signaling pathways were present in this cluster such as orthologs of the Ca^2+^ sensor AtCML25 and the Ca^2+^ associated protein kinases AtCPK13.

#### Cluster R4

Cluster R4 (counter-regulation from down to up) contains in total 394 genes, again with several members of different TF families (Table S4). Interestingly, this cluster contains an ortholog of vascular plant one-zinc finger 1 *(AtVOZ1)*, which has been implicated in heat stress response in plants and acting as a repressor of DREB2C [107]. Cluster R4 also encompasses genes related to glutathione metabolism and detoxification, including four orthologs of the glutathione transferase *AtGSTU18*, for which orthologs were also found in cluster L2 and R2, and three for *AtGSTF13.* Many genes encoding for phi (GSTF) and tau (GSTU) glutathione transferases are upregulated under environmental stress and Arabidopsis plants overexpressing *VvGSTU13* showed enhanced tolerance to a variety of abiotic stress conditions like cold and salt [108]. This cluster contains further anti-oxidant enzymes, including three orthologs of *AtPRX52*, all of them encoded by barley paralogs different from those present in clusters L2, R1, and R2. Cluster R4 exhibits the largest number of HSPs, most of which were small HSPs (SHSPs) as well as HSPs mapping to the Arabidopsis orthologs *AtHSP81-1*, *AtHSP101*, and *AtHSP70*. Also in this cluster we found 14 genes related to photosynthesis.

### Transcription factors as key regulators of Ca^2+^-dependent H_2_O_2_-responsive genes in barley

**Fig. 8:**
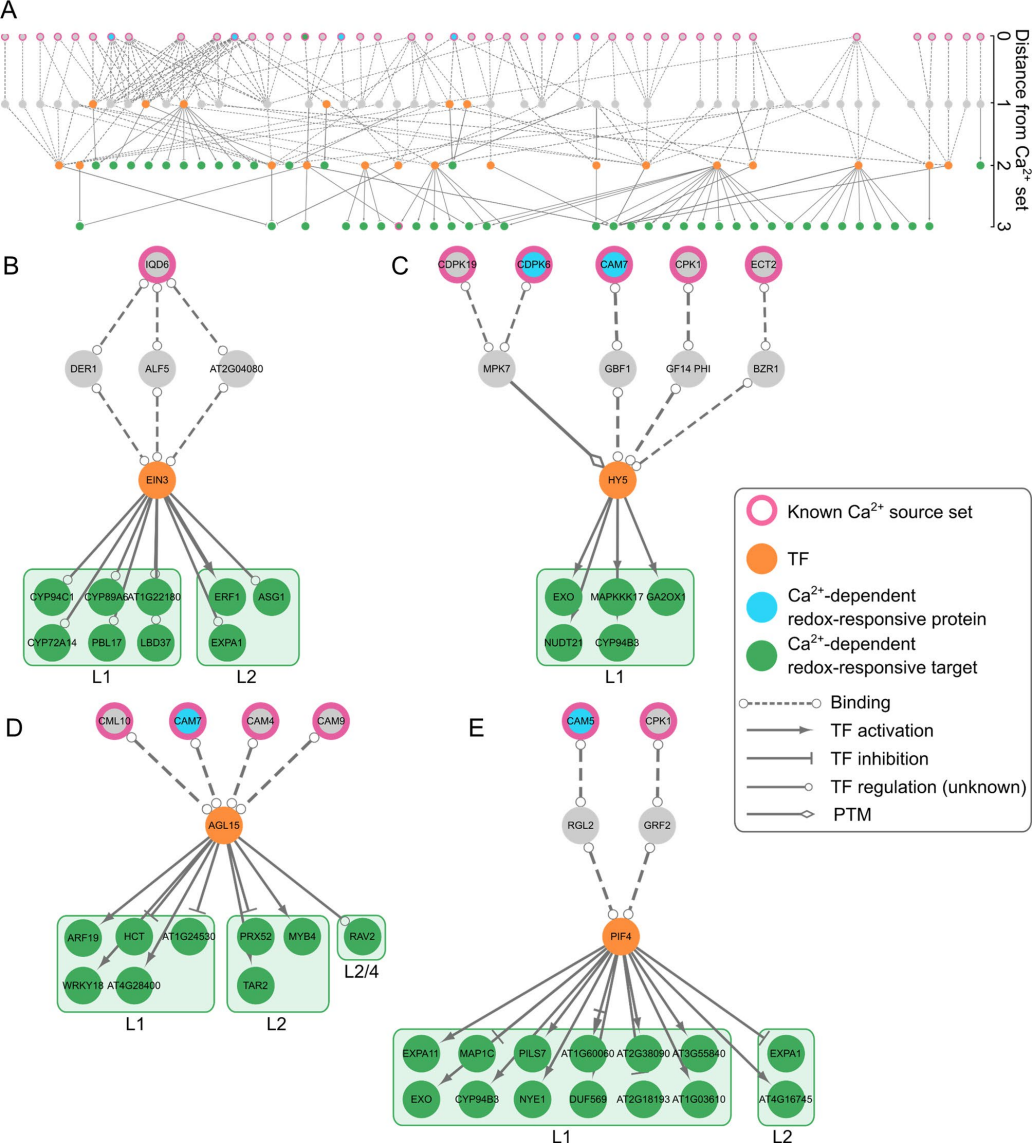
Network analyses of Arabidopsis orthologs of the Ca^2+^-dependent H_2_O_2_-responsive genes found in barley leaves. (**A**) All shortest paths identified in CKN starting from known Ca^2+^-related genes (sources, pink-bordered nodes) to Ca^2+^-dependent H_2_O_2_-responsive genes identified by RNA-seq (targets, green-filled nodes) merged into a single network. Sub-networks were extracted from the merged network with focus on (**B**) EIN3, (**C**) HY5, (**D**) AGL15 and (**E**) PIF4. Ca^2+^-related components identified in a previous proteomic study as H_2_O_2_-regulated in Arabidopsis leaves [65] are presented by a light blue-filling. Nodes are labelled with their short names, when available. The targets are ordered by corresponding clusters (L). PTM: post-translational modification, TF: transcription factor. Complete networks are provided in additional file 1

**Fig. 9:**
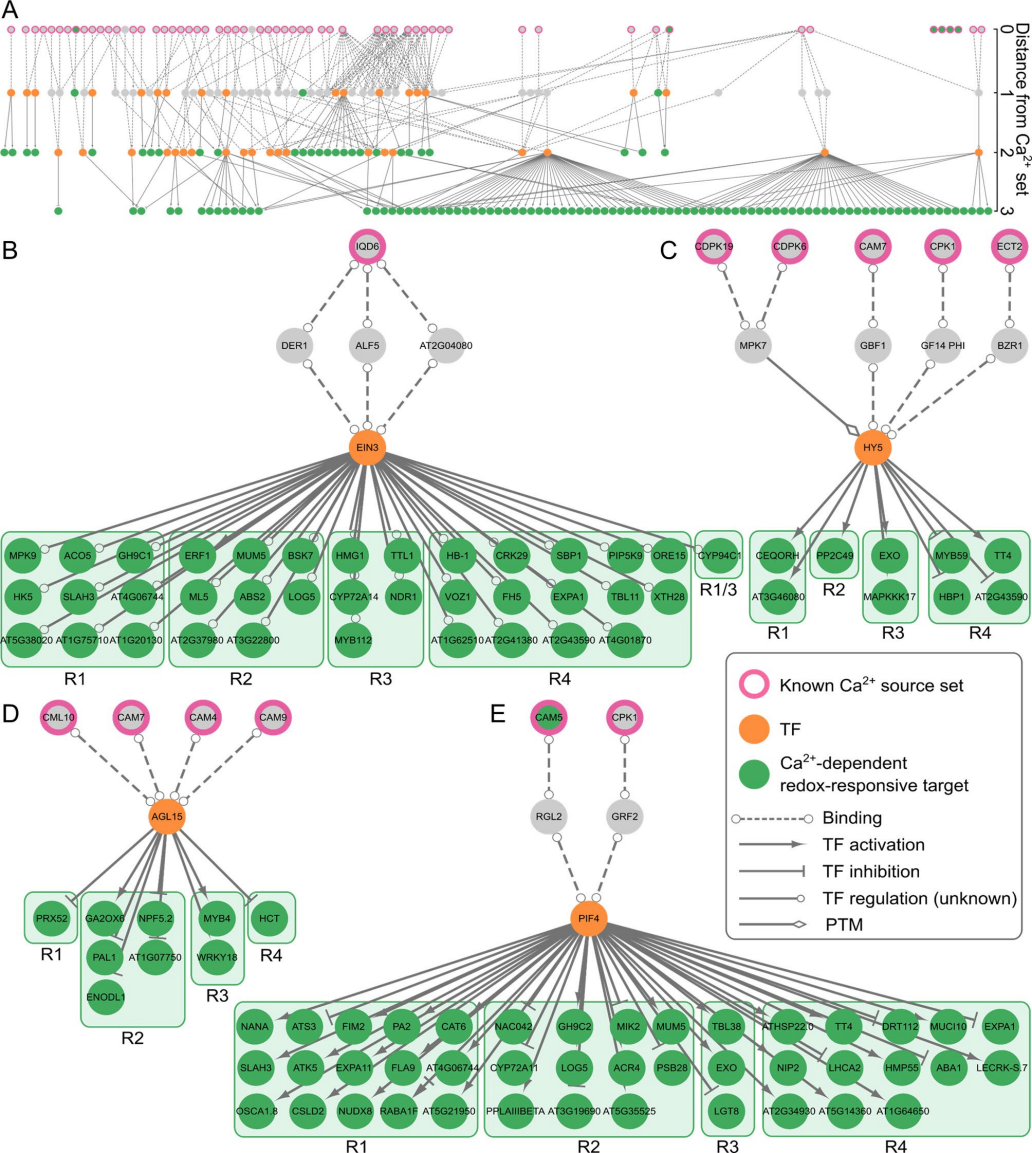
Network analyses of Arabidopsis orthologs of the Ca^2+^-dependent H_2_O_2_-responsive genes found in barley roots. (**A**) All shortest paths identified in CKN starting from known Ca^2+^-related genes. (sources, pink-bordered nodes) to Ca^2+^-dependent H_2_O_2_-responsive genes identified by RNA-seq (targets, green-filled nodes) merged into a single network. Sub-networks were extracted from the merged network with focus on (**B**) EIN3, (**C**) HY5, (**D**) AGL15 and (**E**) PIF4. Nodes are labelled with their short names, when available. The targets are ordered by corresponding clusters (R). PTM: post-translational modification, TF: transcription factor. Complete networks are provided in in additional file 1

We next modelled potential connections from known components of Ca^2+^signaling networks to the identified Ca^2+^-dependent H_2_O_2_-responsive genes (Fig. S4) using CKN of the recently described SKM resource [65]. The information in the CKN is based on present knowledge from Arabidopsis, thus only 192 and 894 Ca^2+^-dependent H_2_O_2_-responsive genes found in leaves and roots of barley, respectively, with identifiable orthologs in Arabidopsis were considered for analysis (Tables S3 and S4). We extracted the directed shortest paths from known Ca^2+^ signaling related genes (source set) to the Ca^2+^-dependent H_2_O_2_-responsive genes identified in our transcriptomic analysis (target set). We additionally required that the final edge regulating the target gene was a transcriptional regulatory interaction. Merging of the results revealed several major network hubs connecting multiple Ca^2+^ signaling components to multiple targets in leaves and roots (Figs. 8A and 9A). The most dominant of these hubs (by number of times they occur in a path as well as number of targets) were depicted separately (Figs. 8B-E and 9B-E). In both, leaves and roots these hubs were defined by the TFs *AGL15*, *HY5*, *PIF4*, and *EIN3* as key nodes regulating several targets (Figs. 8 and 9, orange nodes). The Ca^2+^ signaling components in these networks were mostly CaMs/CMLs and CDPKs/CPKs but also CaM-interacting proteins such as IQD6.

#### Ethylene insensitive 3 (EIN3)

Downstream of *EIN3*, the targets in both tissues include a unique mosaic of genes from different signaling pathways (Figs. 8B and 9B), with a greater prevalence of genes from cluster L1 in leaves (strict positive dependence on cytosolic Ca^2+^ signals) whereas in roots the target genes were interspersed from all the clusters. Noteworthy is the *ERF1* gene, encoding an AP2/ERF transcription factor, which is present in our data as a down-stream target of EIN3 in both tissues (Figs. 8B and 9B). This is in line with a previous study that identified *ERF1* as a downstream component of the ethylene signaling pathway, whose expression is regulated by EIN3 binding to the *ERF1* promoter in vivo [109]. *ERF1* was shown to integrate JA and ethylene signalling pathways in a synergistic manner during plant defense [80] This crosstalk fits to other EIN3-regulated targets found in our dataset such as the JA catabolic protein CYP94C1 and the ethylene biosynthetic protein 1-aminocyclopropane 1-carboxylate oxidase 5 (ACO5), which is known to have EIN3 binding sites [110].

#### Hypocotyl 5 (HY5)

All downstream targets of HY5 in leaves belong to cluster L1 (Fig. 8C), thereby suggesting a pre-dominant strict dependency on Ca^2+^ signals for up-regulation, while in roots this TF again had downstream targets in all clusters (Fig. 9C). The targets in leaves include genes like the MAPKK kinase *MAPKKK17*, involved in plant herbivory responses [111], the phosphatase PP2C49, a negative regulator of salt stress tolerance in Arabidopsis [112], the ceQORH protein, a long-chain fatty acid reductase whose allocation between cytosol and chloroplasts is depending on CaM-binding [113], and the TF MYB59 already established in negative regulation of Ca^2+^ signaling and homeostasis [114]. HY5 is known to play a role in plant thermomorphogenesis in coordination with another TF, *PIF4* [115], which is also present in our network as a nodal hub (see below).

#### Agamous like 15 (AGL15)

Again, the largest group of AGL15 downstream targets in leaves include genes from cluster L1 and L2 (Fig. 8C), representing a strict dependence on Ca^2+^ signals. In roots, the targets of AGL15 include mostly genes from cluster R2 (Fig. 9C), thereby also showing strict dependency on Ca^2+^. Common between leaf and root targets is the TF *MYB4*, which has an established role in protection against oxidative stress during cadmium stress [116] and flavonoid biosynthesis [117]. The targets also include an ortholog of the peroxidase *PRX52*, which has a number of orthologs in barley and is present in different clusters.

#### Phytochrome interacting factor 4(PIF4)

The downstream targets of PIF4, also called *SRL2*, in leaves include mostly genes from cluster L1 (strict dependence on Ca^2+^ signals for H_2_O_2_ induced up-regulation), most of them without a direct relationship to ROS, Ca^2+^ signalling or stress. In roots, downstream targets were found in all clusters and included genes encoding for the Ca^2+^ channel OSCA1.8 involved in osmotic stress induced Ca^2+^ signatures [118], the RAB GTPase RABA1f involved in salt stress response [119], and the TF NAC042 previously shown to be involved in salt and drought stress [120, 121]. Furthermore, targets of PIF4 include genes coding for proteins involved in detoxification of ROS.

## Discussion

Our comparative analysis between the already published transcriptome changes induced by H_2_O_2_ [55] and those observed under a combined application of H_2_O_2_ + LaCl_3_ (this study) showed that the H_2_O_2_-induced Ca^2+^ signals affected the transcript abundance of many H_2_O_2_-responsive genes. The transcriptome changes were not due to an interference with Ca^2+^ homeostasis per se, since only those genes from the H_2_O_2_ + LaCl_3_ set that displayed changes under H_2_O_2_ alone but no changes with LaCl_3_ alone were considered. Overall, in roots more H_2_O_2_-responsive genes showed a dependency on the H_2_O_2_-induced Ca^2+^ signals compared to those in leaves (Fig. 3). This is in line with the higher number of genes for which transcriptional changes were observed after H_2_O_2_ treatment alone in roots [55]. However, even considering these differences in total numbers, expression of only 33% of the H_2_O_2_-responsive genes in leaves, but about 70% of those in roots, was affected by LaCl_3_-sensitive Ca^2+^ signals (Fig. 3B). Moreover, most of the identified Ca^2+^-dependent H_2_O_2_-responsive genes were found only in one of the two tissues, indicating a clear tissue specificity of the response. H_2_O_2_ is not only generated in response to biotic attacks but also by imbalances in energy metabolism. Obviously, photosynthesis is a process generating a large amount of ROS and thus, leaf tissue simply might have a higher prevalence of detoxification systems already in place while they need to be induced upon the accumulation of H_2_O_2_ in roots. This would be in line with the observation that many genes related to oxidative stress and detoxification were observed in response to H_2_O_2_ in roots [55]. We also observed minor differences in H_2_O_2_ penetration (Fig. 1B) and a slightly stronger inhibition of the Ca^2+^ signal (Fig. 1C) by LaCl_3_ in roots which might further affect the transcriptome changes.

**Fig. 10:**
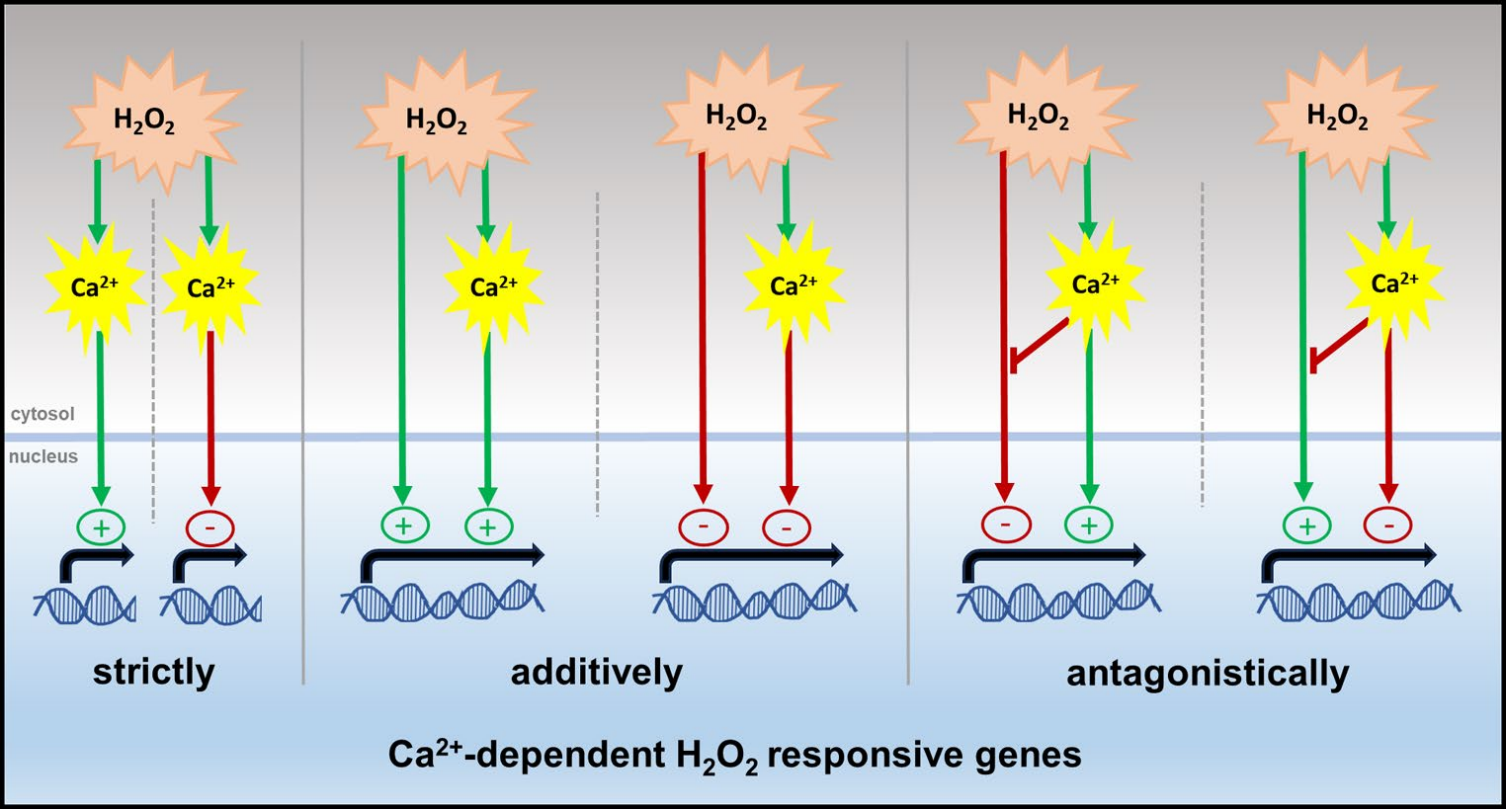
Representative models of Ca^2+^-dependent H_2_O_2_-responses. Strict Ca^2+^-dependency means that Ca^2+^ signaling operates down-stream of H_2_O_2_ to induce either activation or repression of gene expression. Partial dependency is seen when H_2_O_2_ and Ca^2+^ signals modulate gene expression in an additive way. In that case, the H_2_O_2_ activation/repression of gene expression is not fully dependent on the H_2_O_2_-induced Ca^2+^ transient, but Ca^2+^ amplifies this regulation. In the antagonistic model, the H_2_O_2_-induced Ca^2+^ transient inhibits the H_2_O_2_-induced activation/repression while at the same time inducing an opposite response. Lack of the H_2_O_2_-induced Ca^2+^ transient thus results in a changes of transcript abundance from up to down and vice versa. The arrowheads indicate activation (green) or repression (red) and the red T-headed arrows indicate inhibition

The issues discussed above notwithstanding, strict and partial/antagonistic Ca^2+^ dependency of the H_2_O_2_-responsive transcriptome was observed in both tissues (Figs. 3 and 5). Strict dependency (clusters L1, L2, R1, and R2) means that genes with significant changes in transcript level upon H_2_O_2_ application no longer showed significant changes after LaCl_3_ pre-incubation when compared to the control. The most likely scenario for these genes is that a Ca^2+^ signal evoked by H_2_O_2_ is required to activate a transcription activator or repressor (Fig. 10, strictly). This can occur either more directly, e.g., by proteins such as Ca^2+^-dependent TFs or CAMTAs [122], or as the consequence of a longer signalling cascade that involves Ca^2+^ activated proteins such as CDPKs, CaMs, or CBLs [123, 124]. Such strictly Ca^2+^-dependent H_2_O_2_-responsive genes were strongly dominant in leaves (~ 90%) and also the majority in roots (~ 60%). Partially dependent genes showed a difference in transcript abundance between control and H_2_O_2_ + LaCl_3_ treatment; however, the abundance was significantly different from H_2_O_2_ treatment alone. Of these cases, genes in cluster L3 showed a reduced up-regulation in the absence of an H_2_O_2_-induced Ca^2+^ transient, while genes in cluster L4 show reduced down-regulation (Fig. 10, additive). Interestingly, this kind of additive regulation of H_2_O_2_ and Ca^2+^ was completely absent in roots. For genes in these clusters H_2_O_2_ affects changes in transcript abundance both independently and via a Ca^2+^ signal, and both regulations occur in the same direction. Even in the absence of the H_2_O_2_-induced Ca^2+^ transient, the direct regulation by H_2_O_2_ remains. More complex is the regulation of those genes from cluster L5, R3, and R4, for which inhibition of the H_2_O_2_-induced Ca^2+^ transient results in changes of transcript abundance from up to down and vice versa. The regulation of these genes can be explained by an antagonistic model (Fig. 10, antagonistic), where Ca^2+^-dependent and independent pathways act in the opposite direction and Ca^2+^ signaling in addition inhibits or attenuates the Ca^2+^-independent H_2_O_2_ induced activation/repression. Similarly, three genes in cluster L5 that show an increased reduction in transcript abundance in the absence of the Ca^2+^ transient could be regulated by multiple pathways in a Ca^2+^-dependent and -independent manner; however, in this case Ca^2+^ signaling attenuates the H_2_O_2_ response, so that it becomes stronger in its absence (Fig. S5). It should be noted that for all clusters more complex models can be envisioned. Also, transcript abundance is not necessarily defined by gene expression, however, the models can easily be adapted for changes in transcript stability or degradation.

Indeed, our results reinforce the notion of complex, interacting pathways that define the ultimate response to a certain stimulus. While the responses are specific with regards to many factors such as type of stimulus, timing, tissue or developmental stage, they are variances of very similar patterns. After stimuli perception, the information is forwarded through the cell by signaling cascades involving components such as secondary metabolites, ions like Ca^2+^, hormones, kinases, etc., to ultimately affect gene transcription, translation and/or protein activity. The latter is either due to novel synthesis, degradation or alteration of activity that catalyses the molecular changes required. This cascade of event allows for multiple points of regulation and ensures a cross-talk of signals coming from different internal and external stimuli. Many of the intermediate players will be ready and in place to receive a stimulus; nevertheless, stimulus-induced transcriptional regulation of sensors, signaling kinases or TFs can occur to enhance the response or to initiate priming and long-term adaptation. Thus, it is not surprising, that TFs were found in all clusters. It is not uncommon to have TF cascades, in which an initially activated TF affects the transcription of multiple other TFs [125]. Also, different stresses can lead to binding of the target from TFs of different gene families to induce or repress the expression, e.g. the redox‑related LEA protein *SAG21* binding to ERF (pathogen stress), WRKY (H_2_O_2_ stress), and NAC (wounding stress) TF [126].

Phytohormones have been repeatedly demonstrated to interact with each other at various points through versatile TF families, thereby eliciting a common, synchronized, and holistic change in the molecular and biochemical landscape of the plant in response to diverse stimuli [127]. Moreover, the study of interactions between phytohormones and secondary messengers like Ca^2+^ has gained momentum over the years; particularly the CDPKs have been closely linked to phytohormones such as GA, ABA, or JA in regulating crucial plant processes related to growth and development, flowering, and also responses and acclimation to a variety of biotic and abiotic stresses [128]. Other kinases, such as RLKs, were proposed to play crucial roles during growth-defense trade-off, i.e. by intermingling with different phytohormone signal transduction pathways [129]. The presence of these kinases in different clusters is thus in line with a differential regulation through Ca^2+^ signals, but also suggest them as potential hubs which have the potential to transduce downstream signals crucial to the H_2_O_2_-Ca^2+^ crosstalk by interacting with other major signaling pathways like phytohormones.

There are several remarkable differences between the response of leaves and roots. In general, the roots show a higher variety of GO terms compared to leaves (Tables S3 and S4). As mentioned above, roots show more changes in genes related to oxidative stress and detoxification. This is marked by a strong Ca^2+^-dependent regulation of class III peroxidases with a total of 42 peroxidases present across all clusters. Also genes belonging to the GO term cell wall are more abundant in roots compared to leaves. Cell wall metabolism plays important roles in shaping plant responses to stress acclimation [130]. Several reactions associated with crosslinking of cell wall components, like hemicellulose and xyloglucans, along with crucial processes, like polymerization and depolymerization of cell walls, have previously been related to ROS production and anti-oxidant enzyme activities, which is a characteristic feature when plants are challenged with abiotic stress conditions [131]. For instance, the transcription factor short root (SHR) is involved in plant organogenesis including periclinal division in the root cortex that depends on an optimal H_2_O_2_ balance. On one hand, SHR activates H_2_O_2_ production by RBOHs and on the other hand induces SA signaling that increases H_2_O_2_ levels by repressing CATALASE 2 [132].

In roots, we also found a much larger and diverse group of membrane transporters as in leaves, i.e. the wall-associated-transporter-1-like (WAT1) and SWEET-type transporters, but also aquaporins. Aquaporins have been shown to be involved in dynamic ROS changes under stresses [133] and WAT1 was identified as a downstream target of RBOH-mediated ROS generation during parasitic infections [134]. More surprisingly, we could identify 20 genes involved in photosynthesis including LHC proteins and photosystems components to be regulated by H_2_O_2_-induced Ca^2+^ signals in roots. The presence of photosynthesis-related genes in roots might seem a controversial result, but it could be hypothesized that the exposure of the roots to light for five days lead to such a phenomenon. Moreover, it was also proposed that root plastids might be involved in the process of anti-oxidative damage control under stress conditions which generate oxidative bursts [135, 136]. This has also been suggested in another study based on fluorescence spectra of Arabidopsis roots that showed a capacity of root plastids to form larger antenna complexes [137]. Our results therefore might point to a crucial and “less-known” role played by the H_2_O_2_-Ca^2+^ crosstalk in the induction of LHC-encoding genes and other genes related to photosynthesis in roots.

In an attempt to decipher the molecular basis of the Ca^2+^-dependency of the H_2_O_2_-induced transcriptional responses, we modelled potential connections between known components of the Ca^2+^-signaling network and the Ca^2+^-dependent H_2_O_2_-responsive genes identified in this study. The Ca^2+^-signaling components in this network included many CaMs, CMLs, and CDPKs, several of which, had been shown in a recent study in Arabidopsis leaves to undergo Ca^2+^-dependent changes in protein level upon H_2_O_2_ application [65] (Fig. 8, light blue nodes). Moreover, the network analyses showed TFs, especially EIN3, AGL15, PIF4, and HY5, down-stream of the Ca^2+^ components as hubs/nodal points regulating multiple Ca^2+^-dependent H_2_O_2_-responsive genes in different clusters in leaves and roots of barley (Figs. 8 and 9). These TFs are known from Arabidopsis to be involved in different physiological and developmental processes including phytohormone signaling and catabolism, photosynthesis, detoxification, cell wall metabolism, and cellular transport. EIN3 is a positive downstream regulator of the ethylene signalling pathway that affects various facets of plant development, several stress responses, and phytohormone pathways [138]. So far, ethylene signaling involving EIN3 has been related to Ca^2+^ and H_2_O_2_ during salt stress response in Arabidopsis [139]. According to our model, this H_2_O_2_-Ca^2+^ regulation might be mediated by the CaM-binding protein IQD6 (IQ67 Domain Containing 6) (Figs. 8B and 9B), which is known to play a crucial role in plant growth and development [140]. HY5 is a bZIP type master transcriptional regulator of photomorphogenesis, also shown to be involved in other processes such as response to abiotic stresses [141]. It was also shown that HY5 participates in ROS homeostasis [142, 143] and to interact with CAM7 to regulate Ca^2+^-dependent photomorphogenesis in plants [144]. Indeed, in our network CAM7 is connected to HY5 via the G-box-binding factor GBF1(Figs. 8C and 9C), which was shown to play a role in plant defense upstream of SA [145]. We also obtained a connection with CDPK7 and MPK7, which possibly regulate *HY5* expression through post-translational modifications. H_2_O_2_ was also shown to directly increase kinase activity of MPK7, underscoring the complexity of the signaling cross-talk [146]. AGL15 is a member of the MADS box TF family and was shown in vitro to bind CaM [147]. This is in line with our network analyses suggesting connections between AGL15 and multiple CaMs as well as CML10 (Figs. 8D and 9D). As for HY5, AGL15 regulation might also be controlled by CAM7.

PIF4, a member of the bHLH TFs family, has so far very little association with Ca^2+^ and ROS signaling, although a recent report showed a connection to RBOHD-mediated up-regulation under salt stress [148]. RBOH is not present in our model since it was only shown that *PIF4* expression is attenuated in a *rboh* mutant. However, our model suggests regulation of PIF4 by CAM5 and CPK1, which have never been shown to be involved in any stress signaling pathways. Downstream, CAM5 and CPK1 were connected to RGL2 (RGA-Like2), which is a member of the DELLA protein family and has previously been shown to be involved in ROS generation and phytohormonal signaling [149–151]. GRF2 is a member of the 14-3-3 protein family. Although specific data linking GRF2 to signaling or stress pathways is missing, 14-3-3 proteins have been previously linked to plant stress, Ca^2+^ signaling, and hormone signal transduction [152, 153].

However, it should be noted that the information in CKN used for our network modelling is based on current knowledge from Arabidopsis, so only those barley Ca^2+^-dependent H_2_O_2_-responsive genes with identifiable orthologs in Arabidopsis were considered for analyses. Thus, of the 331 and 1334 Ca^2+^-dependent H_2_O_2_-responsive genes in leaves and roots of barley, respectively, only 192 and 894 genes were used in CKN analyses. This clearly reinforces that there is an urgent need for more experimental data to be obtained from barley and other crops to close this vast knowledge gap. While multiple responses are conserved between different land plants, others are more specific. We will need to know the specific responses of crops for accurate stress for modeling and to use this information for improved crop breeding.

## Conclusion

H_2_O_2_ is an indispensable ROS, which is generated as a toxic by-product of biological metabolic processes, but also functions as a signaling molecule that can influence plant growth and development. Moreover, it has an established potential to intermingle with signaling cascades associated with second messengers like Ca^2+^. In this study, using transcriptomic analysis, the molecular landscape behind the tissue-wide H_2_O_2_-Ca^2+^ crosstalk in the crop species barley was elucidated. Our data expands the knowledge on stress response in barley but also strengthen the relevance of findings in model plants such as Arabidopsis for barley. They reveal genes which have never been implicated in any canonical stress response pathway, and therefore may be used as candidates in future studies to further expand our understanding of this crosstalk. Similarly, network analyses suggested nodal TFs which in turn regulate the expression of genes involved in phytohormone pathways including ethylene, JA, ABA, SA, brassinosteroids, GA, and auxin, as well as in MAPK signaling cascades. Several studies have reported that both, biotic and abiotic stress, can lead to the accumulation of H_2_O_2_ and fluctuations in Ca^2+^ levels which imply an enhancement in the vitality of plants to withstand those environmental stress. Hence, deciphering the molecular mechanisms underlying the H_2_O_2_-Ca^2+^ crosstalk will ultimately provide more understanding of stress acclimation not only in barley but also in other crop species.

## Electronic supplementary material

Below is the link to the electronic supplementary material.

Supplementary Material 1: **Additional File 1:** Raw cytoscape output sessions (.cys) of the SKM network analyses in the roots and leaves of barley. Figures 8, 9 and S5 were prepared from the cytoscape files

Supplementary Material 2: **Additional File 2: Fig. S1** Unique and overlapping DEGs between H_2_O_2_ + LaCl_3_ and LaCl_3_ treatment alone vs. control treatment. Venn diagram of DEGs (FDR < 0.01) from (**A**) leaves and (**B**) roots. Only the unique DEGs from the H_2_O_2_ + LaCl_3_ treatment were used for further analyses. **Fig. S2** Determining the number of clusters for Ca^2+^-dependent H_2_O_2_-responsive genes in (**A**) leaves and (**B**) roots. Gap statistics analysis was used for the calculation, with a total of 100 iterations. set.seed(123) function was used before running this function to reduce randomness and inconsistencies in the number of clusters generated. The number of clusters predicted by this analysis was used to perform k-means clustering analyses in figure 5. **Fig. S3** Validation of RNA-seq results by RT-qPCR. Linear regression analysis between transcript level ratios derived from RNA-seq and RT-qPCR data under different treatments in leaves (**A and B**) and roots (**C and D**). C: correlation coefficient, P: P-value, R2: R-regression coefficient. **Fig. S4** CKN analysis of H_2_O_2_ signaling based on Arabidopsis orthologs of the genes identified in barley. All paths identified in CKN leading from known Ca^2+^-involved genes (pink-bordered nodes) to Ca^2+^-dependent H_2_O_2_ responsive genes (green nodes), and from known redox-related genes (blue-bordered nodes) to Ca^2+^-independent H_2_O_2_-responsive genes (yellow nodes), obtained by RNA-seq, merged into a single network in (**A**) leaves and (**B**) roots. Transcription factors are indicated as orange nodes. Complete networks are provided in additional file 1. **Fig. S5** Two potential models for an increased reduction in transcript abundance in the absence of the H_2_O_2_-induced Ca^2+^ transient. This could either occur by a regulation of Ca^2+^-dependent and -independent pathways, which act in opposite directions with different strength of regulation (left panel). Alternatively, the H_2_O_2_-induced Ca^2+^ signals might attenuate the H_2_O_2_ response, so that it becomes stronger in its absence. The arrowheads indicate activation (green) or repression (red)

Supplementary Material 3: **Additional File 3: Table S1** List of primer sequences used for RT-qPCR analyses in this study. Wherever applicable, the corresponding Arabidopsis orthologs are indicated in brackets

Supplementary Material 4: **Additional File 4: Table S2:** Differentially expressed genes (DEGs) between either H_2_O_2_+LaCl_3_ or LaCl_3_ and control samples. Differential expression analysis was carried out with the genes using DESeq2. Attached here are the output files obtained after comparing LaCl_3_ +H_2_O_2_ treated samples with control samples, in the leaf and the root, along with the DESeq2 output files obtained after comparing LaCl_3_ treated samples. DEGs were identified based on adjusted FDR < 0.01 and are listed separately for leaves and roots. Further genes with FDR> 0.01, were considered as genes with unchanged expression (UCs) compared to control samples. Furthermore, the genes commonly regulated between H_2_O_2_+LaCl_3_ and LaCl_3_ treatments were excluded to obtain the genes which are unique for H_2_O_2_+LaCl_3_ treatment and presented separately for leaves and roots of barley

Supplementary Material 5: **Additional File 5: Table S3** A comparison of the obtained unique genes in Leaf H_2_O_2_+LaCl_3_ (Table S2) to leaf H_2_O_2_-DEGs obtained in our former study (Bhattacharyya et al 2023). For the shared genes between both treatments a selection based on a log_2_FC difference (delta log_2_FC) was performed and only genes showing a delta >1 was considered for further analyses. A clustering of the obtained genes resulted in a total of five clusters. control: ddH_2_O; UCs: genes with unchanged expression vs control

Supplementary Material 6: **Additional File 6: Table S4** A comparison of the obtained unique genes in root H_2_O_2_+LaCl_3_ (Table S2) to root H_2_O_2_-DEGs obtained in our former study (Bhattacharyya et al 2023). For the shared genes between both treatments a selection based on a log_2_FC difference (delta log_2_FC) was performed and only genes showing a delta >1 was considered for further analyses. A clustering of the obtained genes resulted in a total of five clusters. control: ddH_2_O; UCs: genes with unchanged expression vs control

## Data Availability

Raw RNA-sequencing data used in this study are available in the SRA (Sequence Read Archive) repository from NCBI (https://www.ncbi.nlm.nih.gov/sra/PRJNA1061386 and https://www.ncbi.nlm.nih.gov/sra/PRJNA973626). Code for the network analyses is available on GitHub (https://github.com/NIB-SI/skm-h2o2-ca2-barley).
