# Supplementary material for: Ca2+-dependent H2O2 response in roots and leaves of barley - a transcriptomic investigation
Source: BMC Plant Biol. 2025 Feb 20;25:232. doi: 10.1186/s12870-025-06248-9 (PMC11841189; doi:10.1186/s12870-025-06248-9)
Supplement: Supplementary file 3 — Supplementary Material 3: Additional File 3: Table S1 List of primer sequences used for RT-qPCR analyses in this study. Wherever applicable, the corresponding Arabidopsis orthologs are indicated in brackets [file 12870_2025_6248_MOESM3_ESM.pdf]

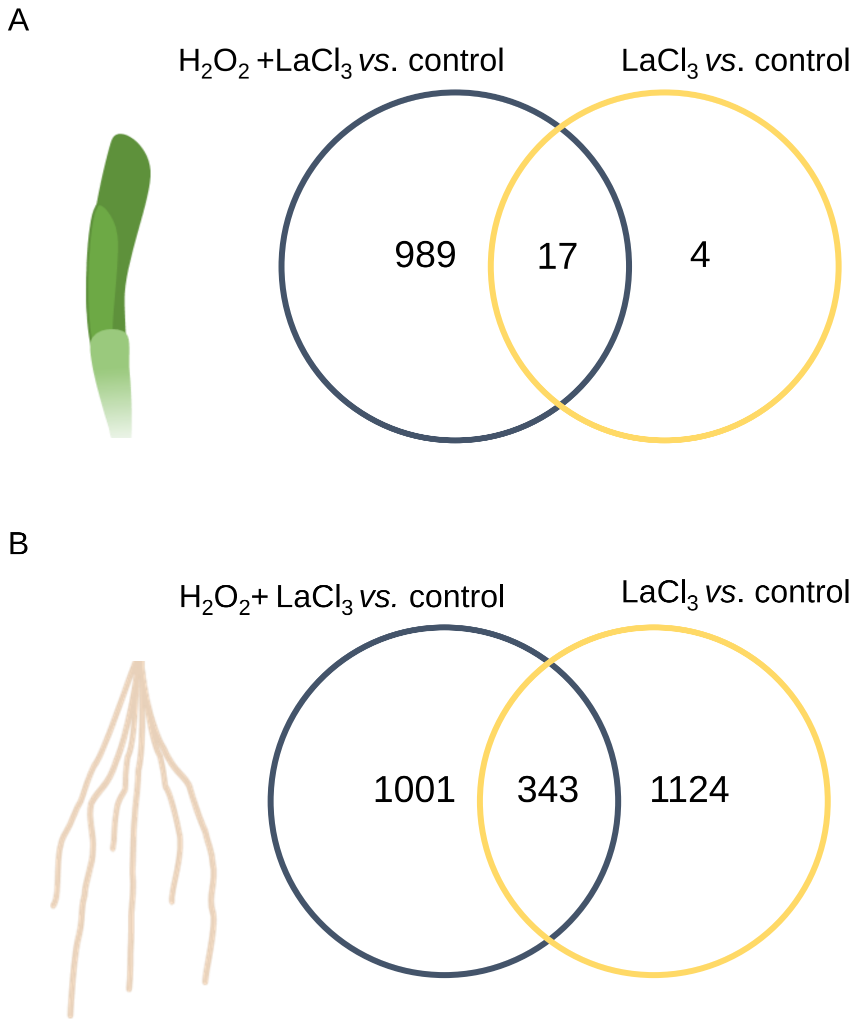

Fig. S1 Unique and overlapping DEGs between H_2O_2+$LaCl_3$ and $LaCl_3$ treatment alone *vs*. control treatment. Venn diagram of DEGs (FDR<0.01) from **(A)** leaves and **(B)** roots. Only the unique DEGs from the H_2O_2+$LaCl_3$ treatment were used for further analyses.

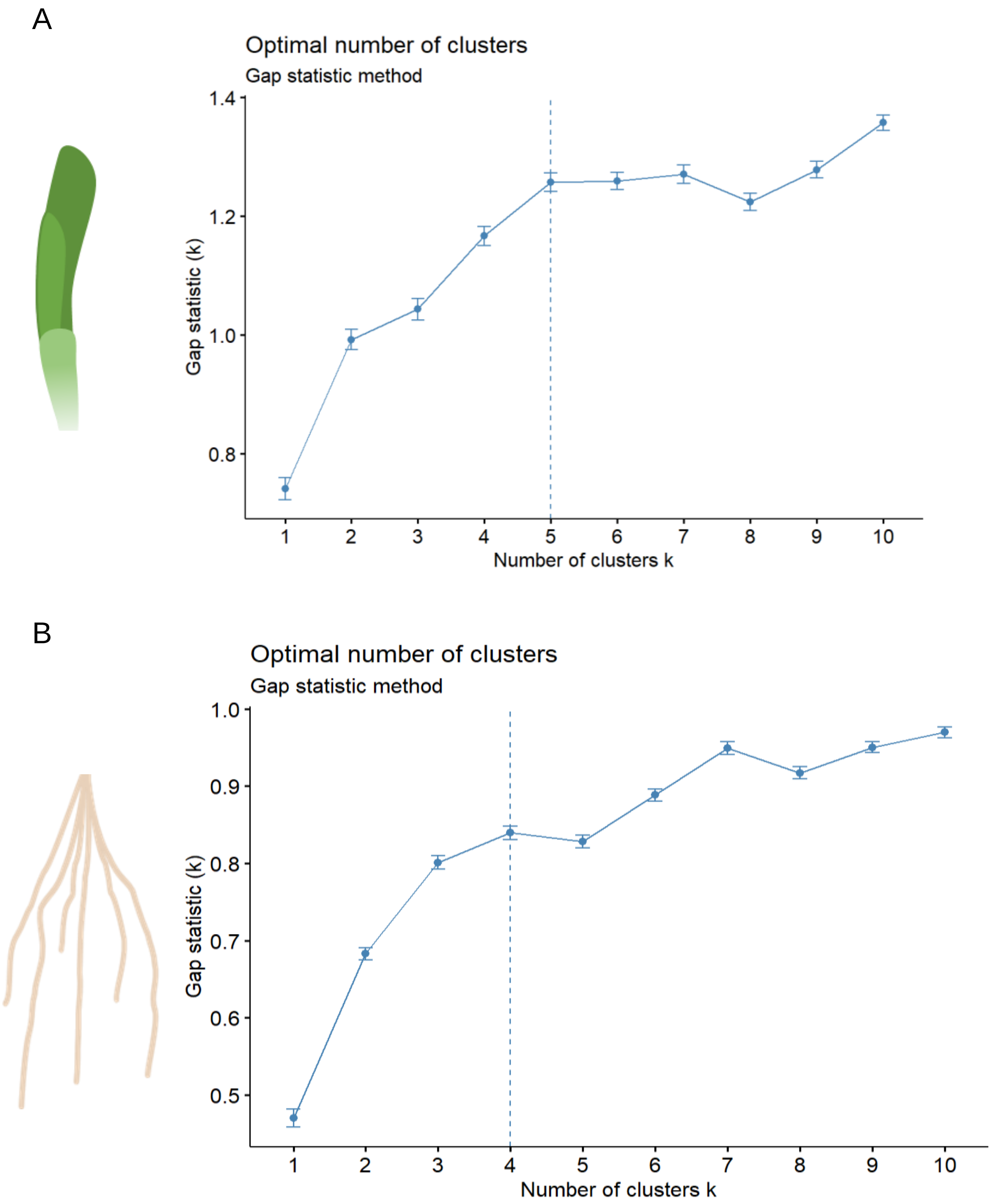

Fig. S2 Determining the optimal number of clusters for Ca^{2+}-dependent H_2O_2-responsive genes in **(A)** leaves and **(B)** roots. Gap statistics analysis was used for the calculation, with a total of 100 iterations. set.seed(123) function was used before running this function to reduce randomness and inconsistencies in the number of clusters generated. The number of clusters predicted by this analysis was used to perform k-means clustering analyses in figure 5.

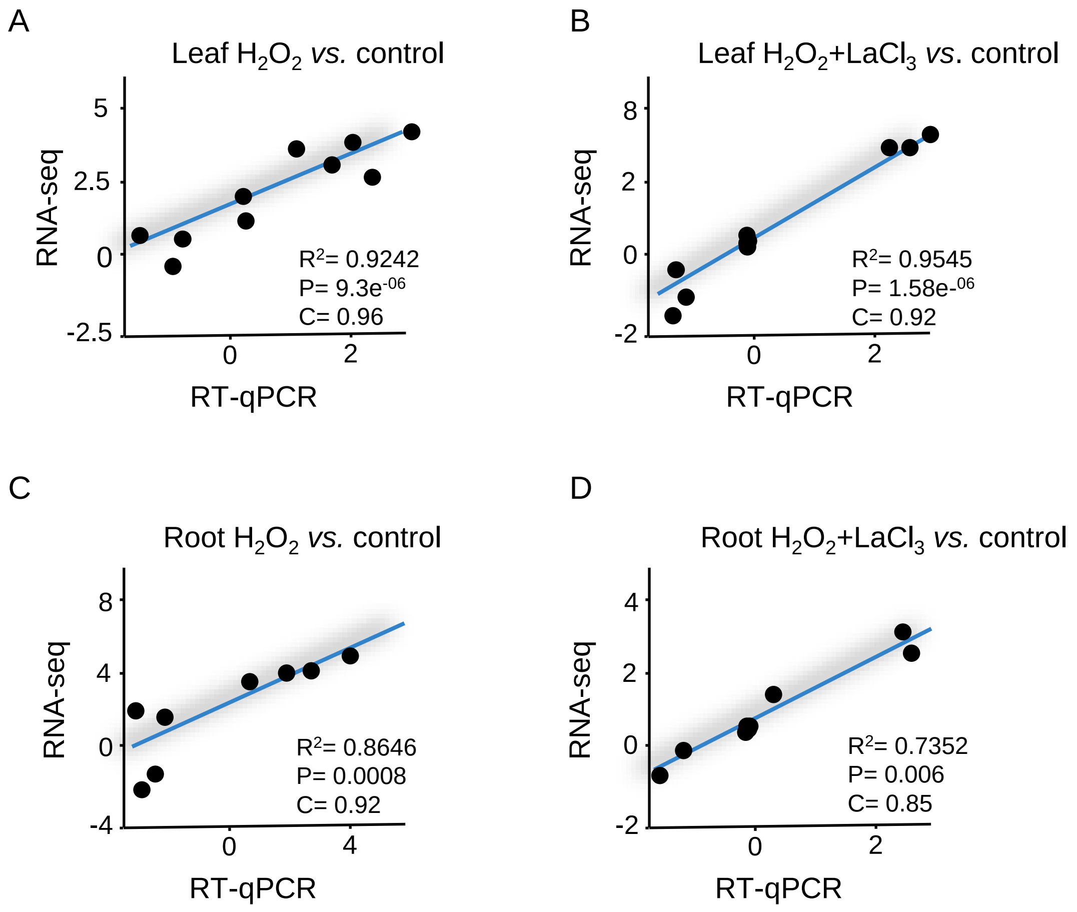

Fig. S3 Validation of RNA-seq results by RT-qPCR. Linear regression analysis between transcript level ratios derived from RNA-seq and RT-qPCR data under different treatments in leaves **(A and B)** and roots **(C and D)**. C: correlation coefficient, P: P-value, R2: R-regression coefficient.

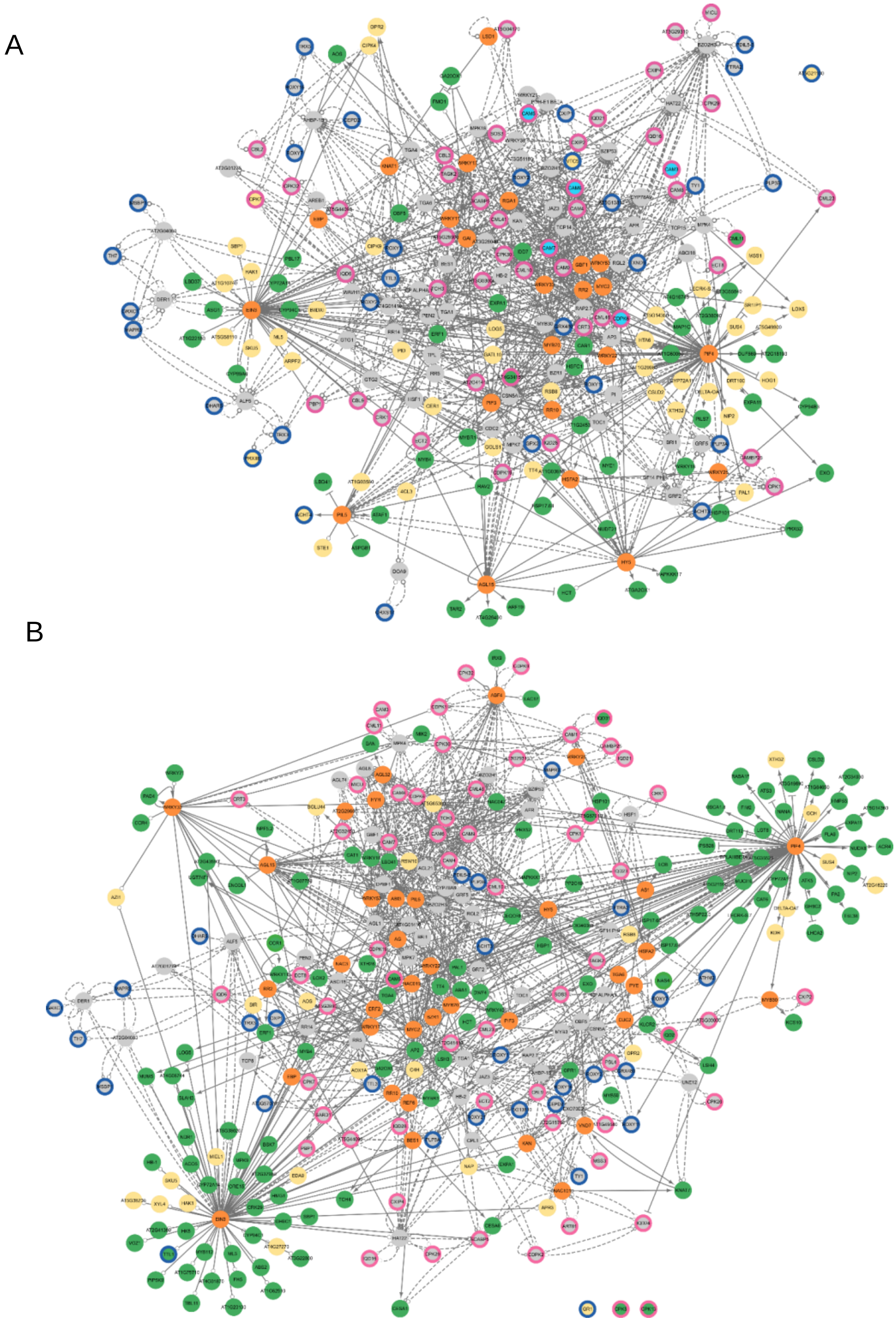

Fig. S4 CKN analysis of H_2O_2 signaling based on Arabidopsis orthologs of the genes identified in barley. All paths identified in CKN leading from known Ca^{2+}-involved genes (pink-bordered nodes) to Ca^{2+}-dependent H_2O_2 responsive genes (green nodes), and from known redox-related genes (blue-bordered nodes) to Ca^{2+}-independent H_2O_2-responsive genes (yellow nodes), obtained by RNA-seq, merged into a single network in **(A)** leaves and **(B)** roots. Transcription factors are indicated as orange nodes. Complete networks are provided in additional file 1.

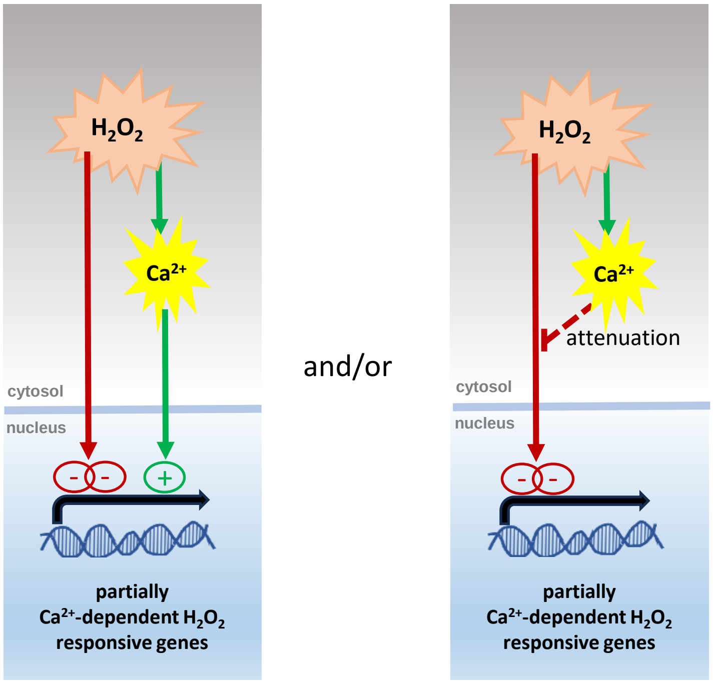

Fig. S5 Two potential models for an increased reduction in transcript abundance in the absence of the H_2O_2-induced Ca^{2+} transient. This could either occur by a regulation of Ca^{2+}-dependent and -independent pathways, which act in opposite directions with different strength of regulation (left panel). Alternatively, the H_2O_2-induced Ca^{2+} signals might attenuate the H_2O_2 response, so that it becomes stronger in its absence. The arrowheads indicate activation (green) or repression (red).
